# Comparative Analysis of the Efficacy and Safety of Different Traditional Chinese Medicine Injections in the Treatment of Cancer-Related Pain: A Bayesian Network Meta-Analysis

**DOI:** 10.3389/fphar.2021.803676

**Published:** 2022-02-07

**Authors:** Pengli Su, Yuanyuan Leng, Jun Liu, Yanan Yu, Zhong Wang, Haixia Dang

**Affiliations:** ^1^ Institute of Basic Research in Clinical Medicine, China Academy of Chinese Medical Sciences, Beijing, China; ^2^ China Academy of Chinese Medical Sciences, Beijing, China

**Keywords:** traditional Chinese medicine injections, analgesics, cancer-related pain, network meta-analysis, efficacy, safety

## Abstract

**Background:** Given the limitations of three-step analgesic therapy and the extensive use of traditional Chinese medicine injections (TCMIs) for cancer-related pain (CRP), this network meta-analysis (NMA) aims to compare the efficacy and safety of different regimens of TCMIs for CRP.

**Methods:** A literature search was conducted in seven electronic databases for all related articles published before 12 April 2021. Randomized controlled trials (RCTs) were screened by a prior eligible criteria. The quality of literature was evaluated by the Cochrane risk of bias tool. We used Stata 16.0 software to analyze data including total pain relief rate, quality of life, and the incidence of adverse reactions. The surface under the cumulative ranking curve (SUCRA) probability values were applied to rank the interventions. Radar map was used to exhibit the most outstanding regimen for a certain outcome. Synthetic sorting bubble diagram was performed to show the relatively better regimen by integrating two or three outcomes.

**Results:** A total of 84 RCTs involving 8,044 patients were included. The results indicated that YDZYR + AN (Yadanziyouru injection plus analgesic) ranked first for pain relief rate, closely followed by KLT + AN (Kanglaite injection plus analgesic). AD + AN (Aidi injection plus analgesic) ranked first for quality of life, KLT + AN following closely. The total adverse reaction rate of FFKS + AN (Fufangkushen injection plus analgesic) was the lowest, and the constipation rate of FFKS was the lowest. In terms of the incidence of nausea and vomiting, KLT + AN was the best choice. In the plots analysis, the results of integrated total incidence of adverse reactions and pain relief rate analysis indicated that FFKS + AN was the most appropriate regimen. Meanwhile, it had the lowest incidence of integrated constipation, nausea and vomiting, and total adverse reactions. KLT + AN was the best in alleviating pain and improving quality of life integrated outcomes.

**Conclusion:** In conclusion, FFKS + AN was the best treatment regimen for the pain relief rate and total adverse reaction rate, and it was also the safest regimen for CRP treatment. KLT + AN was the most effective choice. Further, compared with analgesic treatment alone for patients with CRP, TCMIs + AN combination treatment strategies are significantly more effective. However, more high-quality RCTs are required to support these conclusions.

**Systematic Review Registration:** (https://www.crd.york.ac.uk/prospero/#recordDetails, https://www.crd.york.ac.uk/prospero/export_details_pdf.php), identifier (ChiCTR-ONC-CRD42021267829)

## 1 Introduction

Pain is one of the most common symptoms of cancer, and cancer related pain (CRP) refers to the pain associated with cancer or cancer treatment (Swarm et al., 2019; Chen et al., 2020; Fan et al., 2021; Carr et al., 2002). According to a global cancer statistic from the International Agency for Research on Cancer (IARC), an estimated 19.3 million new cancer cases occurred in 2020 (Sung et al., 2021). Around 75–90% of cancer patients experienced different levels of pain, and approximately 25% was newly diagnosed cancer patients, 33% was undergoing treatment, and up to 75% was advanced cancer patients (Cohen et al., 2003; Goudas et al., 2005; Svendsen et al., 2005; Swarm et al., 2010; Running and Seright, 2012). For cancer patients with metastasis, pain is a common symptom and its incidence is up to 80% (Running and Seright, 2012). In China, the incidence of CRP is 57.4% (Science Popularization Department of Chinese Anti-Cancer Association). Not only does CRP reduce the treatment compliance, but harm physical and mental health of patients, resulting in a heavy burden to the society (Cassileth et al., 2007).

At present, the mainstay of treatment for CRP is the three-step analgesic therapy proposed by the World Health Organization (WHO) in 1986 (Anekar and Cascella, 2021; Ventafridda et al., 1985). It suggests the treatment based on the intensity of pain, from acetaminophen or nonsteroidal anti-inflammatory drug (NSAID) for mild pain (step 1) to morphine-like drugs (step III) for moderate or severe pain (Corli et al., 2016). Although the pain can be controlled to a certain extent, analgesic would produce obvious adverse reactions, drug resistance, or addiction that can sometimes make the original therapy discontinuous or adjusted (Chen et al., 2020; Mercadante, 2015; Corli et al., 2019). A recent study showed that the opioid regimen of 6% of CRP patients had adjusted due to adverse reaction “constipation” (Manuel et al., 2021). Therefore, new regimens with high efficacy and low adverse reactions are of urgent clinical need.

Traditional Chinese medicine injections (TCMIs), as an important component of modern proprietary Chinese medicine, have been widely applied to multiple diseases, especially cancer (Wang et al., 2019; Wu et al., 2019). In clinical practice, CRP is often treated with TCMIs combined with chemical drugs. So TCMIs plus three-step analgesic treatment strategies have been applied for CRP and showed a better efficacy and lower adverse reactions (Lv et al., 2020).

Until April 2021, 22 TCMIs approved by National Medical Products Administration (NMPA) have explicitly mentioned the indications of cancer or CRP in their drug instructions. Through articles literature analysis, we found six kinds of TCMIs have been reported for the treatment of CRP (the detailed TCMI selection process was described in Supplementary Additional File S1). In an expert consensus statement, FFKS (Fufangkushen injection) and HCS (Huachansu injection) have been “A” recommendation as Class I evidence, and KLT (Kanglaite injection) has been “B” recommendation as Class Ⅱ for CRP (Fan et al., 2021). However, no research yet comprehensively compares the efficacy and safety of TCMIs plus analgesics regimes for the treatment of CRP.

Bayesian network meta-analysis (NMA), an approach to combine direct and indirect comparison, has the advantage to compare multiple regimens (Lumley, 2002; Migliore et al., 2012). In view of lacking direct comparisons between different regimens of TCMIs in our study, we applied NMA to evaluate the efficacy and safety of different regimens of TCMIs for CRP. Also, we wanted to assess the necessity of combined treatment of TCMIs and analgesic, which can provide some evidence for the selection of prescription and medical decision-making.

## 2 Methods

This study was reported strictly according to the standard format of the Preferred Reporting Items for Systematic Reviews and Meta-Analysis Specification: PRISMA Extension Statement specification (Page et al., 2021). A completed PRISMA checklist was included as Supplementary Additional File S2.

### 2.1 Literature Search

The related articles, published before April 12, 2021, had been searched at Seven databases including PubMed, Cochrane Library, Embase, China National Knowledge Infrastructure (CNKI), WanFang Database, the Chinese Scientific Journals Full-text Database (VIP), and the Chinese Biomedical Literature Database (CBM). Additional clinical trial data through other sources were also identified, such as the Chinese Clinical Trial Registry (ChiCTR) (http://www.chictr.org.cn/) and the National Institutes of Health (NIH) U.S. National Library of Medicine (https://clinicaltrials.gov/). We used a search strategy combining MeSH terms with free words. The search terms were composed of CRP, TCMIs, and RCT. The TCMIs that we found were 22, which were approved by NMPA for cancer or cancer pain. The detailed search strategies were described in Supplementary Additional File S3. The protocol of this NMA has been registered at the International prospective register of systematic reviews (CRD42021267829).

### 2.2 Inclusion Criteria

#### 2.2.1 Types of Participants

Patients who suffered from CRP were included. The primary cancer was diagnosed according to the histopathological or cytological examination. Gender, age, and nationality were unrestricted.

#### 2.2.2 Types of Interventions

Patients in treatment group received TCMIs or TCMIs combined with analgesic, while those in control group received analgesic solely.

#### 2.2.3 Types of Studies

RCTs for CRP were eligible, with or without blinding.

#### 2.2.4 Types of Outcomes

Outcomes included the total pain relief rate, quality of life, and adverse reaction rate. The included articles should have one of these efficacy outcomes. The primary outcome was the total pain relief rate. The reduction in pain intensity was measured using a numerical rating scale (NRS), visual analogue scale (VAS), or verbal rating scale (VRS). The main reference criteria for pain relief were as follows (Lv et al., 2020): patients with partial relief or above (≥50%) or with marked effect or above were regarded as effective cases (i.e., the pain was tolerable and did not affect normal life or sleep). The secondary outcome was quality of life which was measured by Karnofsky performance score (KPS). Based on KPS, we divided the quality of life into three levels: improved (KPS increased by more than 10 points), stable (KPS changed by less than 10 points), and decreased (KPS score decreased by more than 10 points). The improved and stable levels were considered as efficacy. The safety outcomes were the total incidence of adverse reactions, nausea and vomiting as well as constipation.

### 2.3 Exclusion Criteria

The exclusion criteria were as follows: 1) There is other TCM treatment except for the above 22 TCMIs in the treatment group (such as acupuncture); 2) The repeatedly published articles or unable to find the outcome data; 3) Researches with incomplete data or obvious errors; 4) Study types were reviews, nonclinical studies, or meta-analysis.

### 2.4 Data Extraction and Quality Assessment

Endnote X 9.1 software was used to manage all retrieved articles. After excluding duplicates, two researchers (PS and YL) independently screened articles according to the inclusion and exclusion criteria. A preliminary screening was carried out based on the title and abstract, and then rescreening was performed by reading the full text. After identifying the eligible studies, the data of articles were extracted using a specially designed form including publication data (publication date, title, and authors’ names), details of patients’ characteristics (sample sizes, age, and sex), interventions (the kinds of TCMIs and analgesic and the course of treatment), outcomes (the primary and secondary outcomes), and factors to evaluate the risk of bias.

Two researchers (PS and YL) independently conducted the quality assessment of all included RCTs according to the risk of bias assessment tool recommended in the Cochrane Handbook 5.1. Each study was assessed as low, high, or unclear risk of bias based on seven quality evaluation items, including random sequence generation, allocation concealment, blinding of participants and personnel, blinding of outcome assessment, incomplete outcome data, selective reporting, and other bias. In case of disagreement between the two researchers during the screening of studies, extraction of data, and evaluation of literature quality, this disagreement was resolved by consensus or by consulting a third researcher (HD). Finally, the results of bias risk assessment were summarized and mapped by using RevMan 5.3 software.

### 2.5 Statistical Analysis

Statistical analyses were performed with Stata16.0 software and Microsoft Excel 2019 software. Odds ratios (OR) or mean differences (MD) with 95% confidence intervals (CIs) were calculated for discrete or continuous data respectively. Since the included RCT differed methodologically and clinically, the random-effects model was conducted in this NMA. Sensitivity analysis was used to assess the robustness of the results. A network diagram of interventions was constructed to show the relationships between regimens. If there was a closed loop of various interventions, an inconsistency test was required to explore the network heterogeneity between direct and indirect comparisons within triangular loops. The results were expressed as *p* value, IF (inconsistency factor), and 95% CIs. The surface under the cumulative ranking area curve (SUCRA) was used to rank the multiple interventions, with SUCRA values of 100 and 0% assigned to the best and worst treatments. The number of iterations set at 5,000. Also, we created a pictorial presentation for all five outcomes *via* a radar map using Microsoft Excel 2019 software. If the intervention exhibited outstanding efficacy relative to other treatments for a certain outcome, it would appear on the outermost side of the corresponding line in the radar map. Furthermore, we utilized synthetic sorting bubble diagram diagrams in two or three dimensions to show the relative better regimen of TCMIs. Interventions located in the upper-right corner were superior to others. Finally, a comparison-adjusted funnel plot was created to assess the publication bias.

The present NMA did not need to require ethical approval because it gathered data from previously published trials.

## 3 Results

### 3.1 Search Results

**FIGURE 1:**
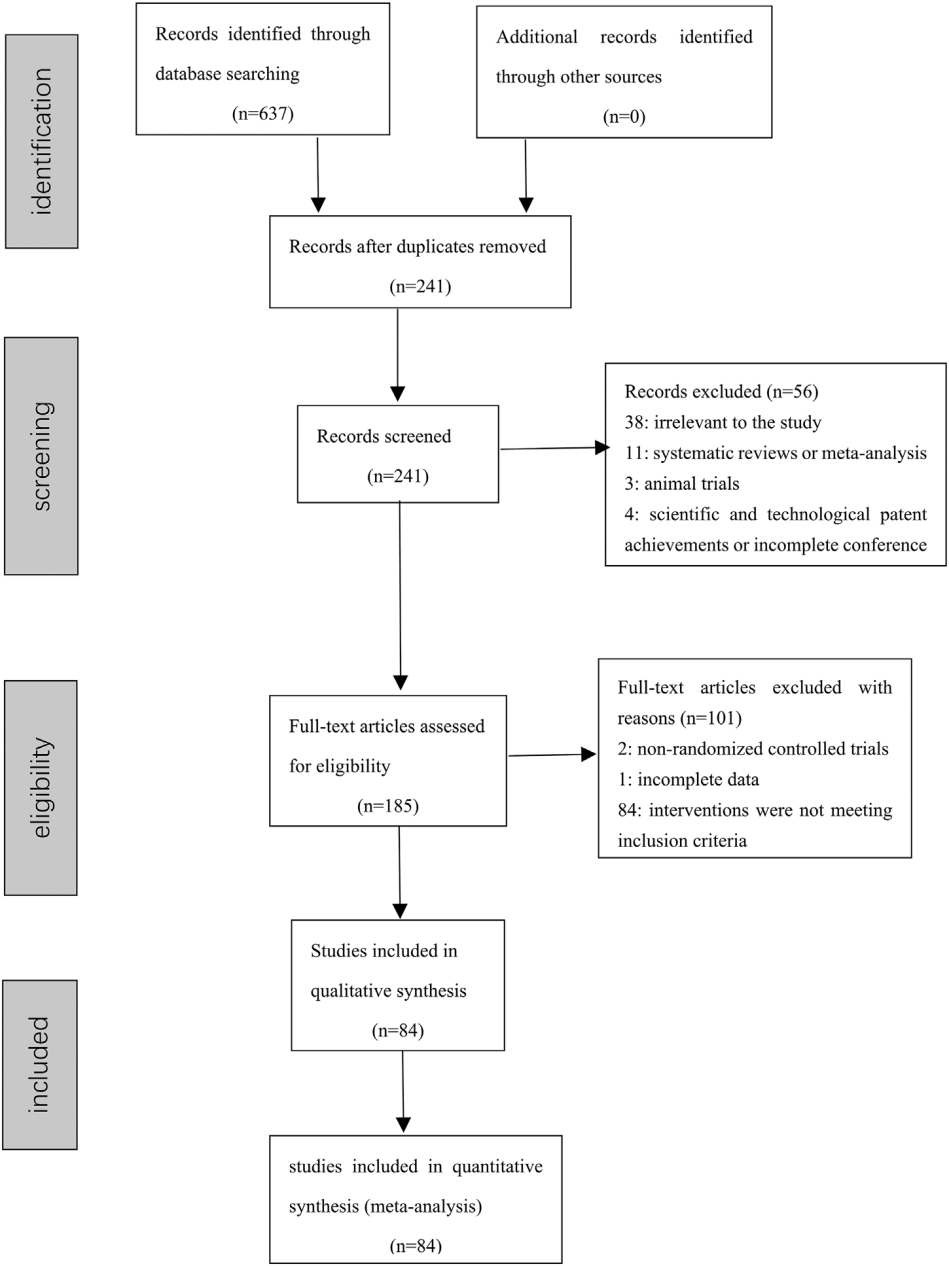
Prisma flow diagram.

Out of the 637 retrieved articles, 84 RCTs were included in the NMA. Further details of the literature screening process were shown in Figure 1.

### 3.2 Basic Characteristics of Included Studies

Overall, 84 studies enrolled 8,044 patients and the largest sample size was 360 and the smallest was 30. A total of 4,040 patients received TCMIs or TCMIs plus analgesic (the treatment group) and 4,004 received analgesic solely (the control group). All the included studies were conducted in China. Only one study was three-arm (Qu, 2015); the others were two-arm. Nine interventions were included in this NMA: FFKS + AN (Fufangkushen injection plus analgesic) (64 RCTs), HCS + AN (Huachansu injection plus analgesic) (5 RCTs), AD + AN (Aidi injection plus analgesic) (4 RCTs), XAP + AN (Xiaoaiping injection plus analgesic) (1 RCT), KLT + AN (Kailaite injection plus analgesic) (3 RCTs), YDZYR + AN (Yadanziyouru injection plus analgesic) (1 RCT), FFKS (4 RCTs), HCS (2 RCTs), and AN (analgesic). The basic information about TCMIs that we concerned about was described in Supplementary Additional File S4. Three types of analgesics were included: the drug commonly used in patients with cancer bone metastasis (zoledronic acid, Yi Ban phosphonic acid sodium), non-steroidal anti-inflammatory and analgesic drugs, and opioid analgesics. The details of the included study characteristics were shown in Table 1 and Supplementary Additional File S5.

**TABLE 1:** Characteristics of the included randomized controlled trials.

Study ID	Sample size (T/C)	Age (year)		Gender (M/F)		Intervention		Course of treatment (days)	Grading of cancer	Outcomes
		T	C	T	C	T	C			
Liu et al. (2005a)	30/76	50.5		69/37		FFKS + AN	AN	10 days	—	1
Liu et al. (2005b)	23/21	66.3	62.8	19/11	20/10	HCS	AN	28 days	mild, moderate, severe pain	1, 2, 3
Zou et al. (2006)	43/40	58.3		51/32		FFKS + AN	AN	7 days	moderate, severe pain	1, 3
Chen et al. (2007)	60/60	57.6		78/42		FFKS + AN	AN	7 days	moderate, severe pain	1, 3
Ma et al. (2008)	60/60	57.6		78/42		FFKS + AN	AN	7 days	—	1, 3
Si et al. (2008)	37/68	57.6 ± 10.7	56.9 ± 11.2	28/9	49/19	FFKS + AN	AN	56 days	—	1, 2, 3
Yang et al. (2009)	38/36	56.7	57.5	45/29	45/27	FFKS + AN	AN	21 days	—	1, 2, 3
Cheng, (2009)	40/36	58.3 ± 3.15	56.0 ± 2.42	25/19	22/14	KLT + AN	AN	21 days	—	1, 2, 3
Su et al. (2009)	32/30	71	70	27/5	25/5	FFKS + AN	AN	20 days	moderate, severe pain	1, 3
Liu et al. (2010)	50/46	52–80		51/45		FFKS + AN	AN	10 days	moderate, severe pain	1, 3
Pan (2010)	40/40	37–76	38–75	21/19	19/21	FFKS + AN	AN	7 days	severe pain	1, 3
Chen (2011)	32/30	71	70	27/5	25/5	AD + AN	AN	20 days	moderate, severe pain	1, 3
Lang (2011)	32/30	71	70	27/5	25/5	AD + AN	AN	20 days	moderate, severe pain	1, 3
Xu et al. (2011)	48/47	45–78		60/38		FFKS + AN	AN	10 days	moderate, severe pain	1, 2, 3
Lin et al. (2011)	52/52	51.7	53.3	32/20	30/22	FFKS + AN	AN	12 days	-	1, 2, 3
Guan and Lin, (2011)	150/150	66.7 ± 5.3	68.3 ± 5.6	85/65	83/67	FFKS + AN	AN	7 days	moderate, severe pain	1, 2, 3
Fu et al. (2012)	60/60	62		74/46		FFKS + AN	AN	7 days	moderate, severe pain	1, 2, 3
Dou and Guo (2012)	31/31	60	61	19/12	18/13	FFKS + AN	AN	12 days	—	1, 2, 3
Chen et al. (2012)	54/48	mild pain:61.5	62.1	14/12	12/12	FFKS	AN	10 days	mild, moderate, severe pain	1, 3
		moderate, severe pain:59.5	60.3	17/11	14/12					
Yang et al. (2012)	30/30	62	58	16/14	18/12	FFKS + AN	AN	10 days	moderate, severe pain	1, 3
Zeng (2011)	50/48	52–88		53/45		FFKS + AN	AN	10 days	Moderate, severe pain	1, 3
Ming (2013)	28/28	46.3 ± 13.2	45.8 ± 11.3	18/10	17/11	FFKS + AN	AN	10 days	—	1, 3
Huang (2013)	50/42	61.5 ± 8.98	32/18	62.1 ± 10.01	26/16	FFKS	AN	7 days	mild pain	1
Zhao (2013a)	30/30	63		29/31		FFKS + AN	AN	7 days	Moderate, severe pain	1, 2, 3
Zhao (2013b)	23/23	63 ± 13	61 ± 14	13/10	11/12	FFKS + AN	AN	14 days	—	1, 3
Yao et al. (2013)	35/35	52.4		52/18		YDZYR + AN	AN	14 days	—	1, 2, 3
Dai (2013)	36/30	62.3 ± 2.8	61.6 ± 2.7	20/16	16/14	FFKS + AN	AN	17 days	—	1, 2, 3
Wang (2013)	32/30	71	70	27/5	25/5	FFKS + AN	AN	20 days	Moderate, severe pain	1, 3
Qi and Du (2013)	82/80	55	57	45/37	51/29	FFKS + AN	AN	28 days	—	1, 2, 3
Cai et al. (2013)	43/42	38–79		49/38		FFKS + AN	AN	14 days	Moderate, severe pain	1, 3
Zhao and Ni (2013)	65/65	68	69	35/30	36/29	FFKS + AN	AN	10 days	—	1, 3
Xie (2013)	45/45	38–75	36–74	22/23	23/22	FFKS + AN	AN	7 days	—	1, 2, 3
Liu (2014)	32/32	57–79		54/10		FFKS + AN	AN	14 days	—	1, 2, 3
Wang and Gao (2014)	50/50	48.8 ± 6.8	45.6 ± 5.6	29/21	26/24	FFKS	AN	10 days	mild, moderate, severe pain	1, 2
Huang and Feng (2014)	37/37	50–78	47–76	20/17	21/16	FFKS + AN	AN	14 days	—	1, 3
Li et al. (2014)	60/60	52.3	53.4	38/22	40/20	FFKS + AN	AN	14 days	moderate, severe pain	1, 2, 3
Zhang (2014)	45/45	55.12 ± 5.21	56.21 ± 5.19	25/20	30/15	FFKS + AN	AN	28 days	—	1, 2, 3
He et al. (2014)	57/56	56		70/48		FFKS + AN	AN	15 days	moderate, severe pain	1, 2, 3
Zhou and Li (2014)	70/40	45–83	45–84	38/32	23/17	FFKS + AN	AN	20 days	moderate, severe pain	1, 2, 3
Yang et al. (2014)	37/36	67.7	68.1	44/28	42/29	AD + AN	AN	21 days	—	1, 2, 3
Jin et al. (2014)	61/61	53.4 ± 10.4	52.9 ± 9.9	35/26	36/25	FFKS + AN	AN	10 days	—	1, 2, 3
Sun et al. (2014)	35/35	56.5 ± 11.5	61.4 ± 10.7	13/22	15/20	FFKS + AN	AN	14 days	Moderate, severe pain	1, 3
Luan et al. (2014)	45/45	40–72	38–71	24/21	22/23	FFKS + AN	AN	7 days	severe pain	1, 3
Zhang et al. (2015)	37/36	51.8 ± 5.7	51.2 ± 6.1	22/15	21/15	FFKS + AN	AN	14 days	—	1, 3
Zhao (2015)	30/30	63		29/31		HCS + AN	AN	7 days	moderate, severe pain	1, 2, 3
Feng et al. (2015)	30/30	72.5 ± 4.5	71.4 ± 9.7	11/19	13/17	FFKS + AN	AN	14 days	moderate, severe pain	1, 3
Qu (2015)	30/30/30	Low dose group: 55.4	53.2	24/6	23/7	FFKS + AN	AN	14 days	moderate, severe pain	1, 3
		high dose group: 54.7		21/9						
Yuan (2015)	50/50	65	63	28/22	27/23	FFKS + AN	AN	10 days	—	1, 3
Zhang (2015)	35/35	39–80	45–79	21/14	18/17	HCS + AN	AN	7 days	moderate pain	1, 2
Zhang (2016)	45/44	54.8 ± 3.9	56.2 ± 4.4	23/22	21/23	FFKS + AN	AN	15 days	—	1, 2, 3
Zhao (2016)	40/40	62		47/33		FFKS + AN	AN	20 days	moderate, severe pain	1, 2, 3
Wang et al. (2016)	144/144	64.7 ± 10.2		159/129		HCS	AN	14 days	moderate, severe pain	1, 2, 3
Mo et al. (2016)	90/90	46.6 ± 4.1	45.7 ± 3.8	48/42	45/45	FFKS + AN	AN	14 days	—	1, 2
Chang (2016)	100/100	mild pain:48.3 ± 5.1	48.5 ± 2.7	31/19	29/21	FFKS	AN	10 days	mild, moderate, severe pain	1, 2, 3
		moderate pain:47.7 ± 3.7	47.6 ± 3.4	29/21	28/22					
Zhai Z 2017	30/30	48 ± 8	50 ± 9	17/13	16/14	FFKS + AN	AN	28 days	—	1, 2, 3
Chen (2017)	57/57	47.74 ± 10.68	47.01 ± 10.75	30/27	32/25	FFKS + AN	AN	30 days	severe pain	1, 2, 3
Jiang et al. (2017)	12/9	49.5 ± 2.3	48 ± 4	10/5	9/6	HCS + AN	AN	14 days	mild, moderate, severe pain	1, 2
Tian (2017)	32/32	58.6 ± 3.2	58.1 ± 3.7	19/13	17/15	FFKS + AN	AN	14 days	—	1, 3
Lei and Wen (2017)	40/40	61 ± 11	56 ± 14	21/19	20/20	FFKS + AN	AN	28 days	moderate, severe pain	1, 2, 3
Liu et al. (2017a)	34/34	45–75		43/25		FFKS + AN	AN	7 days	moderate, severe pain	1, 2, 3
Liu et al. (2017b)	46/46	77.98 ± 3.65	78.82 ± 3.33	22/24	26/20	FFKS + AN	AN	10 days	—	1, 2, 3
Yan (2017)	50/50	61.3 ± 2.4	61.5 ± 2.6	27/23	29/21	FFKS + AN	AN	14 days	moderate, severe pain	1, 2, 3
Yang et al. (2017)	39/39	28–85	31–86	26/13	24/15	FFKS + AN	AN	7 days	moderate, severe pain	1, 3
Wang et al. (2017)	50/50	71.0 ± 5.9	70.0 ± 6.6	31/19	28/22	FFKS + AN	AN	21 days	moderate, severe pain	1, 2, 3
Long et al. (2017)	42/42	53.7 ± 6.9	53.1 ± 6.7	19/23	20/22	KLT + AN	AN	14 days	—	1, 2, 3
Yan and Zhang, (2018)	33/33	43.21 ± 1.34	42.17 ± 1.66	19/14	18/15	FFKS + AN	AN	10 days	—	1, 3
Wen et al. (2018)	41/41	58.9 ± 5.4	58.7 ± 5.1	25/17	23/18	HCS + AN	AN	14 days	moderate, severe pain	1, 2, 3
Feng et al. (2018)	36/36	66.7 ± 4.12	65.4 ± 3.68	23/13	22/14	FFKS + AN	AN	10 days	—	1, 2, 3
Hao (2018)	33/33	56.3 ± 2.5	57.1 ± 2.1	18/15	19/14	FFKS + AN	AN	7 days	—	1, 3
Li et al. (2018)	49/49	52.06 ± 8.05	51.68 ± 7.94	29/20	28/21	FFKS + AN	AN	10 days	severe pain	1, 2, 3
Xia et al. (2018)	28/28	45–75		40/16		FFKS + AN	AN	7 days	moderate, severe pain	1, 2, 3
Nie (2018)	40/40	54.27 ± 12.76	48.31 ± 9.25	21/19	18/22	FFKS + AN	AN	30 days	—	1, 3
Luo and Lin (2018)	32/32	—	—	—	—	KLT + AN	AN	28 days	moderate, severe pain	1, 2,
Ren et al. (2018)	40/40	59.1 ± 13.8		22/18		FFKS + AN	AN	14 days	—	1
Zhang (2018)	60/60	—	—	34/26	35/25	FFKS + AN	AN	10 days	mild, moderate, severe pain	1, 2, 3
Wang et al. (2018)	120/120	58.65 ± 18.63	57.85 ± 17.87	75/45	73/47	HCS + AN	AN	28 days	—	1, 2, 3
Wei et al. (2018)	180/180	85.05+-5.79	84.96+-5.77	143/37	145/35	FFKS + AN	AN	10 days	—	1, 2, 3
Liu et al. (2019)	40/40	60.56 ± 5.98	61.52 ± 5.23	24/16	23/17	FFKS + AN	AN	7 days	—	1, 2
Dong et al. (2019)	27/27	38–79	36–79	15/12	13/14	FFKS + AN	AN	7 days	moderate, severe pain	1, 2, 3
Long (2020)	25/25	60.21 ± 5.56		21/29		FFKS + AN	AN		—	1
Jiang et al. (2020)	51/51	60.10 ± 9.06	60.45 ± 9.19	23/28	25/26	XAP + AN	AN	14 days	moderate, severe pain	1, 2, 3
Ling (2020)	29/29	53.17 ± 13.48	54.92 ± 13.57	16/13	17/12	AD + AN	AN	7 days	—	1, 2, 3
Fei (2020)	50/50	60.35 ± 8.36	61.57 ± 8.14	30/20	28/22	FFKS + AN	AN	10 days	—	1, 2
Li (2021)	40/40	65.92 ± 5.70	66.08 ± 5.65	22/18	23/17	FFKS + AN	AN	14 days	—	1, 2

Note: T, treatment group; C, control group; NR, not reported; FFKS + AN, Fufangkushen injection + analgesic; HCS + AN, Huachansu injection + analgesic; AD + AN, Aidi injection + analgesic; XAP + AN, Xiaoaiping injection + analgesic; KLT + AN, Kanglaite injection + analgesic; YDZYR + AN, Yadanziyouru injection + analgesic; AN, analgesic; FFKS, fufangkushen injection; HCS, huachansu injection.

Outcome 1. Pain relief rate; 2. Quality of life; 3. Adverse Reactions.

### 3.3 Risk of Bias Assessment

**FIGURE 2:**
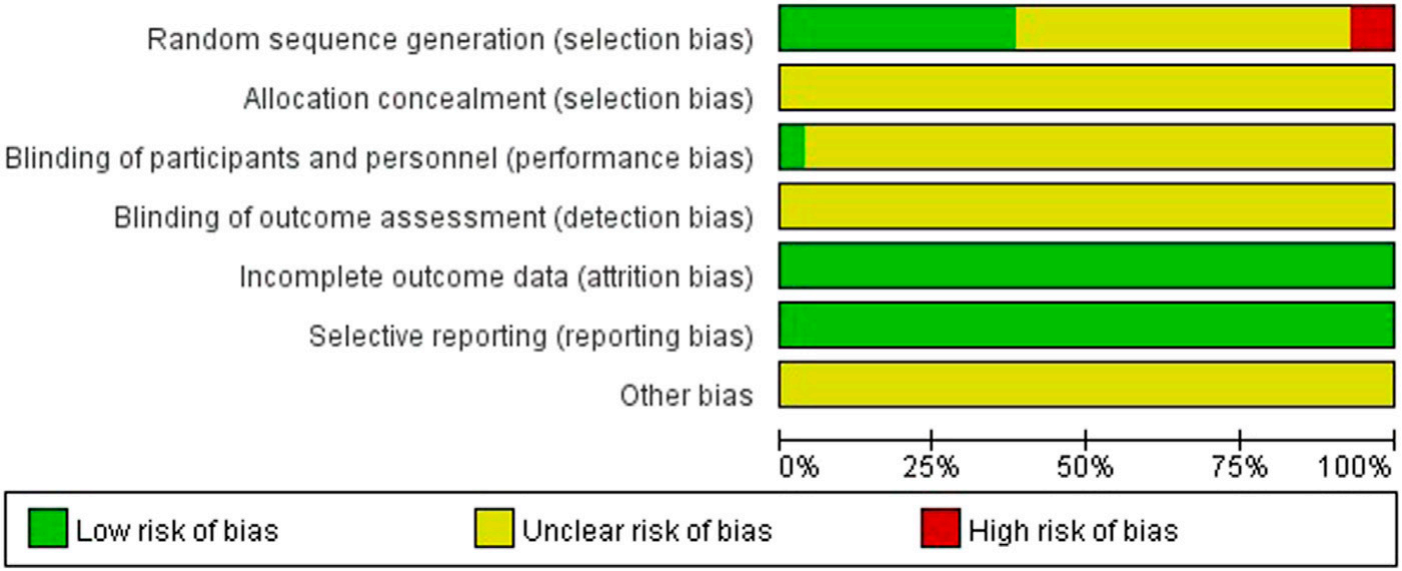
Assessment of risk of bias.

Thirty-one of the 84 studies described appropriate methods for generating random sequences; thus, their selection bias was evaluated as “low risk.” Five studies reported inappropriate methods (randomly in patient’s sequence of entering into the hospital); thus, their selection bias was classified as “high risk.” One study adopted the stratified randomization method (Chang, 2016), and one adopted the complete randomization grouping method (Dou and Guo, 2012), but the specific method of random sequence generation was not clearly explained. The remaining studies only mentioned “random”; thus, the selection bias of these 48 studies was assessed as “unclear.” None of the studies reported the processes used for allocation concealment and blinding of outcome assessment; thus, their bias was considered as “unclear.” Three studies mentioned the blinding of participants and personnel; thus, their bias was assessed as “low risk.” The risk of bias in the remaining literature was rated as “unclear.” All studies had complete data, so the attrition bias was evaluated as “low risk.” All studies reported the outcomes described in their methods section, so their reporting bias was deemed as “low risk.” The other bias risk was rated as “unclear,” because there were no available details to evaluate. All results were shown in Figure 2.

### 3.4 Network Meta-Analysis

#### 3.4.1 Efficacy

##### Total Pain Relief Rate

**FIGURE 3:**
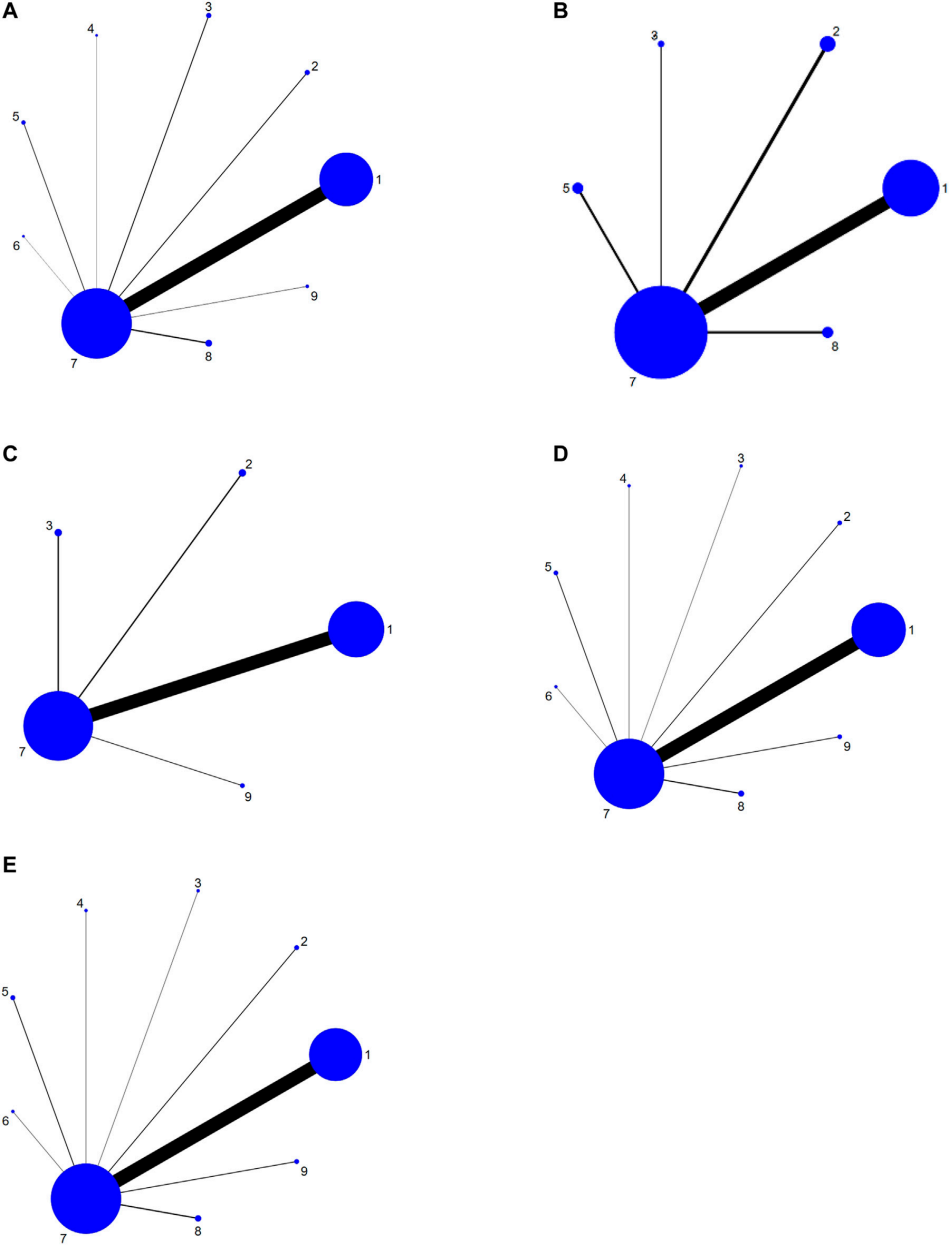
Network graph for different outcomes. **(A)** Pain relief rate; **(B)** KPS; **(C)** Total Adverse Reactions; **(D)** Nausea and vomiting; **(E)** constipation. Note: 1, Fufangkushen injection + analgesic; 2, Huachansu injection + analgesic; 3, Aidi injection + analgesic; 4, Xiaoaiping injection + analgesic; 5, Kanglaite injection + analgesic; 6, Yadanziyouru injection + analgesic; 7, analgesic; 8, Fufangkushen injection; 9, Huachansu injection.

A total of 83 studies referred to the total pain relief rate, involving six TCMIs and nine interventions. There were 64 studies on FFKS + AN, four studies on HCS + AN, four studies on AD + AN, one study on XAP + AN, three studies on KLT + AN, one study on YDZYR + AN, four studies on FFKS, and two studies on HCS. Using analgesic as the comparison, eight pairs direct comparisons were generated, and no closed loop was formed. A network of comparisons between interventions was shown in Figure 3.

Compared with AN solely, FFKS + AN (OR = 0.39, 95% CI [0.34–0.45]), HCS + AN (OR = 0.39, 95% CI [0.24–0.61]), AD + AN (OR = 0.43, 95% CI [0.25–0.75]), XAP + AN (OR = 0.41, 95% CI [0.18–0.93]), KLT + AN (OR = 0.26, 95% CI [0.12–0.56]), and YDZYR + AN (OR = 0.22, 95% CI [0.06–0.77]), HCS (OR = 0.45, 95% CI [0.26–0.77]) could improve the pain relief rate and make the difference between groups statistically significant. Comparing to FFKS alone, FFKS + AN (OR = 0.31, 95% CI [0.20–0.48]), HCS + AN (OR = 0.30, 95% CI [0.16–0.56]), AD + AN (OR = 0.34, 95% CI [0.17–0.68]), XAP + AN (OR = 0.32, 95% CI [0.13–0.81]), KLT + AN (OR = 0.21, 95% CI [0.09–0.50]), and YDZYR + AN (OR = 0.17, 95% CI [0.05–0.65]) were found to have more efficacy in relieving pain. Compared with HCS alone, FFKS + AN (OR = 0.18, 95% CI [0.10–0.31]), HCS + AN (OR = 0.17, 95% CI [0.09–0.35]), AD + AN (OR = 0.19, 95% CI [0.09–0.42]), XAP + AN (OR = 0.18, 95% CI [0.07–0.49]), KLT + AN (OR = 0.12, 95% CI [0.05–0.30]), and YDZYR + AN (OR = 0.10, 95% CI [0.02–0.39]) were even more effective in relieving pain. The OR values were shown in Table 2.

**TABLE 2:** Statistical results of network meta-analysis for efficacy outcomes of the various interventions (ORs, 95% CI).

Intervention	Pain relief rate	KPS
FFKS + AN vs		
HCS + AN	1.01 (0.63, 1.63)	0.60 (0.27, 1.33)
AD + AN	0.90 (0.51, 1.60)	3.49 (0.39, 31.05)
XAP + AN	0.96 (0.41, 2.23)	—
KLT + AN	1.50 (0.68, 3.27)	2.39 (0.93, 6.15)
YDZYR + AN	1.79 (0.50, 6.36)	—
AN	**0.39 (0.34, 0.45)**	**0.33 (0.22, 0.48)**
FFKS	**0.31 (0.20, 0.48)**	0.96 (0.48, 1.92)
HCS	**0.18 (0.10, 0.31)**	—
HCS + AN vs		
AD + AN	0.89 (0.44, 1.82)	5.83 (0.60, 56.31)
XAP + AN	0.95 (0.37, 2.44)	—
KLT + AN	1.48 (0.60, 3.61)	**4.00 (1.31, 12.25)**
YDZYR + AN	1.76 (0.46, 6.75)	—
AN	**0.39 (0.24, 0.61)**	0.55 (0.27, 1.11)
FFKS	**0.30 (0.16, 0.56)**	1.61 (0.65, 4.01)
HCS	**0.17 (0.09, 0.35)**	—
AD + AN vs		
XAP + AN	1.06 (0.39, 2.88)	—
KLT + AN	1.66 (0.64, 4.27)	0.69 (0.07, 7.00)
YDZYR + AN	1.98 (0.50, 7.85)	—
AN	**0.43 (0.25, 0.75)**	**0.09 (0.01, 0.81)**
FFKS	**0.34 (0.17, 0.68)**	0.28 (0.03, 2.57)
HCS	**0.19 (0.09, 0.42)**	—
XAP + AN vs		
KLT + AN	1.56 (0.50,4.84)	—
YDZYR + AN	1.86 (0.41,8.43)	—
AN	**0.41 (0.18,0.93)**	—
FFKS	**0.32 (0.13,0.81)**	—
HCS	**0.18 (0.07,0.49)**	—
KLT + AN vs		
YDZYR + AN	1.19 (0.27, 5.24)	—
AN	**0.26 (0.12, 0.56)**	**0.14 (0.06, 0.33)**
FFKS	**0.21 (0.09, 0.50)**	0.40 (0.14, 1.14)
HCS	**0.12 (0.05, 0.30)**	—
YDZYR + AN		
AN	**0.22 (0.06, 0.77)**	—
FFKS	**0.17 (0.05, 0.65)**	—
HCS	**0.10 (0.02, 0.39)**	
AN vs		
FFKS	0.79 (0.52, 1.20)	**2.95 (1.65, 5.25)**
HCS	**0.45 (0.26, 0.77)**	—
FFKS vs		
HCS	0.57 (0.29, 1.12)	—

Note: Bold results indicate statistically significant differences between groups.

FFKS + AN, Fufangkushen injection + analgesic; HCS + AN, Huachansu injection + analgesic; AD + AN, Aidi injection + analgesic; XAP + AN, Xiaoaiping injection + analgesic; KLT + AN, Kanglaite injection + analgesic; YDZYR + AN, Yadanziyouru injection + analgesic; AN, analgesic; FFKS, fufangkushen injection; HCS, huachansu injection.

**FIGURE 4:**
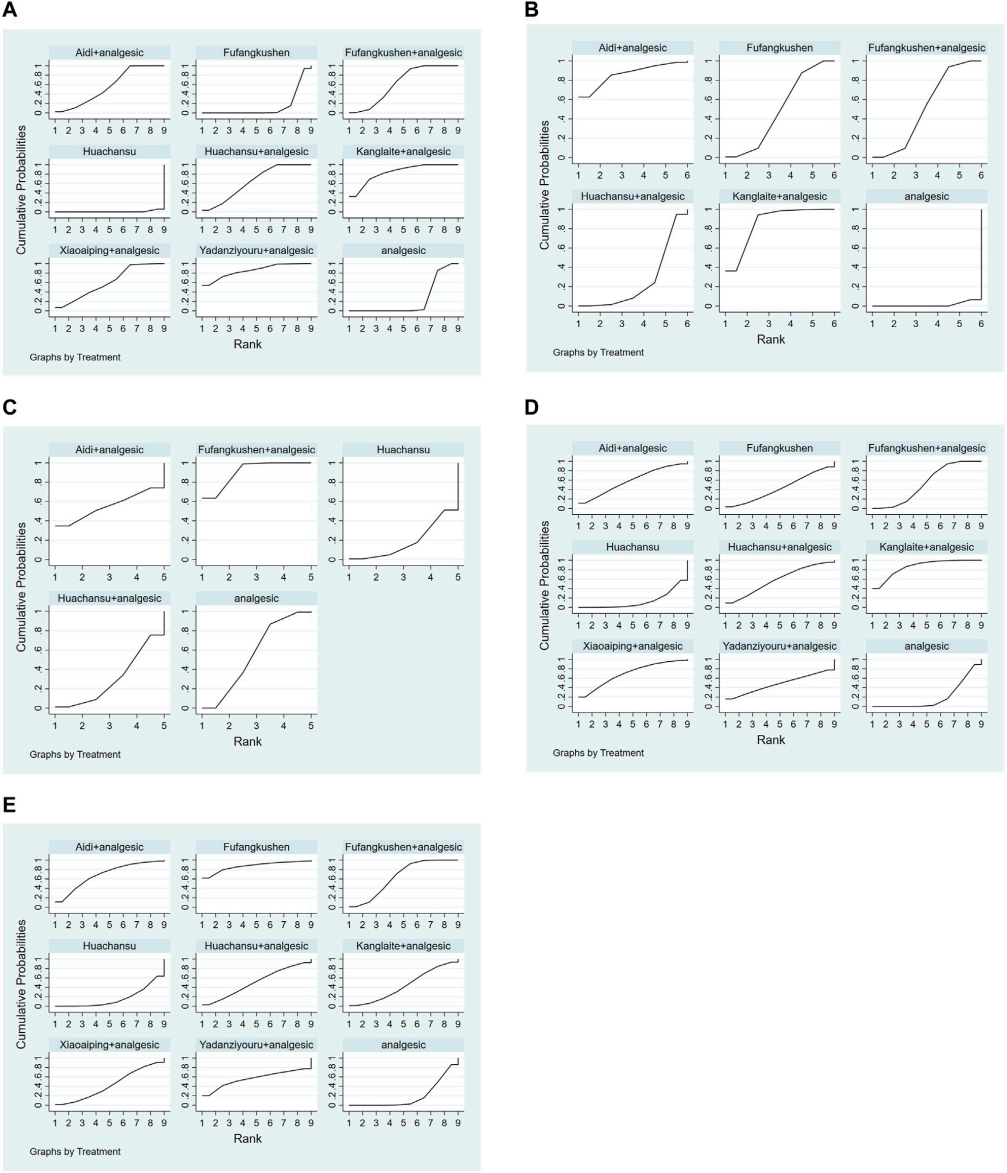
Plot of the surface under the cumulative ranking curves for all treatments. **(A)** Pain relief rate; **(B)** KPS; **(C)** Total Adverse Reactions; **(D)** Nausea and vomiting; **(E)** constipation.

The SUCRA rank and probability value results indicated that YDZYR + AN (85.3%) was the most likely to improve pain relief rate, followed by KLT + AN (83.6%), HCS + AN (63.8%), FFKS + AN (62.6%), XAP + AN (60.4%), AD + AN (56.2%), AN (23.5%), FFKS (13.8%), and HCS (0.7%) (Figure 4; Table 3).

**TABLE 3:** Surface under the cumulative ranking probabilities (SUCRA) results of efficacy outcomes.

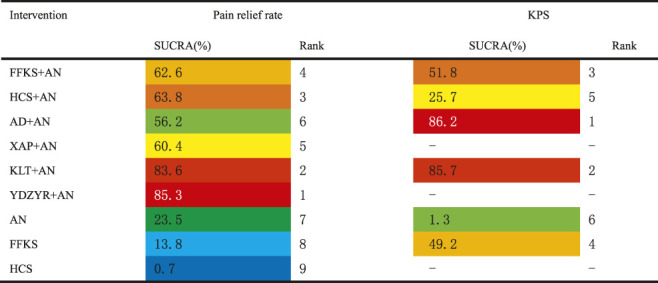

Note: The warmer the color, the greater the SUCRA, and the greater the probability of becoming the best intervention. FFKS + AN, Fufangkushen injection + analgesic; HCS + AN, Huachansu injection + analgesic; AD + AN, Aidi injection + analgesic; XAP + AN, Xiaoaiping injection + analgesic; KLT + AN, Kanglaite injection + analgesic; YDZYR + AN, Yadanziyouru injection + analgesic; AN, analgesic; FFKS, fufangkushen injection; HCS, huachansu injection.

##### Quality of Life

Twenty studies reported the quality of life, which constituted five pairs of direct comparisons, involving four TCMIs and six interventions (FFKS + AN, HCS + AN, AD + AN, KLT + AN, AN, FFKS). The network diagram was shown in Figure 3. Since it did not form a closed loop, no inconsistency test was carried out.

Fifteen pairs comparisons were generated among the six interventions, five of which showed statistically differences. Compared with AN solely, FFKS + AN (OR = 0.33, 95% CI [0.22–0.48]), KLT + AN (OR = 0.14, 95% CI [0.06–0.33]), and AD + AN (OR = 0.09, 95% CI [0.01–0.81]) showed more effective to improve the quality of life. KLT + AN had more efficacy than HCS + AN (OR = 4.00, 95% CI [1.31–12.25]). FFKS was superior to AN (OR = 2.95, 95% CI [1.65–5.25]) in improving the quality of life, as shown in Table 2.

After ranking of six interventions based on the SUCRA values, the results were as follows: AD + AN (86.2%), KLT + AN (85.7%), FFKS + AN (51.8%), FFKS (49.2%), HCS + AN (25.7%), and AN (1.3%), as shown in Figure 4 and Table 3.

#### 3.4.2 Safety

Seventy-four studies mentioned the occurrence of adverse reactions, in which 25 studies reported the total incidence of adverse reactions (7 reported no adverse reaction during treatment). The adverse reactions mainly include dizziness, headache, dysuria, abdominal discomfort, diarrhea, nausea, vomiting, poor appetite, and drowsiness. Among all types of adverse reactions, the most frequent occurrences were nausea and vomiting (48 RCTs) and constipation (45 RCTs). No closed loop was formed in terms of safety outcomes indicators.

##### Total Incidence of Adverse Reactions

In terms of the total incidence of adverse reactions, 25 studies consisted of four pairs comparisons, involving three types of TCMIs and five interventions (FFKS + AN, HCS + AN, AD + AN, AN, HCS). The network diagram was shown in Figure 3.

There were 10 pairs comparisons in the NMA of total incidence of adverse reactions, and three indicated statistically significant differences. Compared with AN (OR = 1.87, 95% CI [1.29–2.72]), HCS + AN (OR = 2.93 [1.10–7.81]), and HCS (OR = 3.89 [1.25–12.07]), FFKS + AN was safer in terms of total incidence of adverse reactions, as shown in Table 4.

**TABLE 4:** Statistical results of network meta-analysis for safety outcomes of the various interventions (OR value, 95% CI).

Intervention	Total adverse reactions	Nausea and vomiting	Constipation
FFKS + AN vs			
HCS + AN	**2.93 (1.10, 7.81)**	0.89 (0.32, 2.45)	1.19 (0.45, 3.18)
AD + AN	1.75 (0.10, 31.81)	0.89 (0.29, 2.73)	0.82 (0.28, 2.40)
XAP + AN	—	0.72 (0.24, 2.13)	1.37 (0.60, 3.12)
KLT + AN	—	0.51 (0.22, 1.16)	1.33 (0.62, 2.88)
YDZYR + AN	—	1.07 (0.17, 6.90)	0.93 (0.08, 10.80)
AN	**1.87 (1.29, 2.72)**	**1.65 (1.34, 2.03)**	**1.91 (1.57, 2.32)**
FFKS	—	1.17 (0.41, 3.31)	0.35 (0.05, 2.28)
HCS	**3.89 (1.25, 12.07)**	**1.93 (1.02, 3.65)**	**2.06 (1.11, 3.80)**
HCS + AN vs			
AD + AN	0.60 (0.03, 12.14)	1.00 (0.23, 4.40)	0.69 (0.17, 2.88)
XAP + AN	—	0.80 (0.19, 3.45)	1.15 (0.33, 4.02)
KLT + AN	—	0.57 (0.16, 2.03)	1.12 (0.33, 3.78)
YDZYR + AN	—	1.20 (0.15, 9.81)	0.78 (0.06, 10.78)
AN	0.64 (0.26, 1.56)	1.85 (0.69, 4.99)	1.60 (0.61, 4.19)
FFKS	—	1.31 (0.32, 5.44)	0.29 (0.04, 2.39)
HCS	1.33 (0.33, 5.36)	2.16 (0.68, 6.90)	1.73 (0.56, 5.31)
AD + AN vs			
XAP + AN	—	0.80 (0.17, 3.72)	1.67 (0.44, 6.25)
KLT + AN	—	0.57 (0.15, 2.22)	1.62 (0.45, 5.89)
YDZYR + AN	—	1.20 (0.14, 10.34)	1.13 (0.08, 16.16)
AN	1.07 (0.06, 18.87)	1.85 (0.62, 5.56)	2.32 (0.81, 6.64)
FFKS	—	1.31 (0.29, 5.88)	0.42 (0.05, 3.61)
HCS	2.22 (0.10, 47.61)	2.16 (0.62, 7.58)	2.50 (0.75, 8.32)
XAP + AN vs			
KLT + AN	—	0.71 (0.19, 2.70)	0.97 (0.33, 2.90)
YDZYR + AN	—	1.49 (0.18, 12.69)	0.67 (0.05, 8.86)
AN	—	2.31 (0.79, 6.73)	1.39 (0.63, 3.09)
FFKS	—	1.63 (0.37, 7.17)	0.25 (0.03, 1.93)
HCS	2.08 (0.71, 6.06)	2.69 (0.79, 9.20)	1.50 (0.56, 4.02)
KLT + AN vs			
YDZYR + AN	—	2.11 (0.28, 15.88)	0.69 (0.05, 8.97)
AN	—	**3.26 (1.46, 7.26)**	1.43 (0.68, 3.01)
FFKS	—	2.31 (0.63, 8.45)	0.26 (0.03, 1.95)
HCS	—	**3.80 (1.39, 10.37)**	1.54 (0.60, 3.97)
YDZYR + AN			
AN	—	1.55 (0.24, 9.88)	2.06 (0.18, 23.83)
FFKS	—	1.10 (0.13, 9.09)	0.38 (0.02, 8.17)
HCS		1.80 (0.26, 12.68)	2.22 (0.18, 27.49)
AN vs			
FFKS	—	0.71 (0.26, 1.96)	0.18 (0.03, 1.18)
HCS	—	1.17 (0.64, 2.14)	1.08 (0.60, 1.93)
FFKS vs			
HCS	—	1.65 (0.50, 5.39)	5.91 (0.84, 41.72)

Note: Bold results indicate statistically significant differences between groups.

FFKS + AN, Fufangkushen injection + analgesic; HCS + AN, Huachansu injection + analgesic; AD + AN, Aidi injection + analgesic; XAP + AN, Xiaoaiping injection + analgesic; KLT + AN, Kanglaite injection + analgesic; YDZYR + AN, Yadanziyouru injection + analgesic; AN, analgesic; FFKS, fufangkushen injection; HCS, huachansu injection.

Based on the SUCRA values, the five interventions were ranked as follows: FFKS + AN (90.6%), AN (55.7%), AD + AN (55.2%), HCS + AN (30%), and HCS (18.6%), as shown in Figure 4 and Table 5.

**TABLE 5:** Surface under the cumulative ranking probabilities (SUCRA) results of safety outcomes.

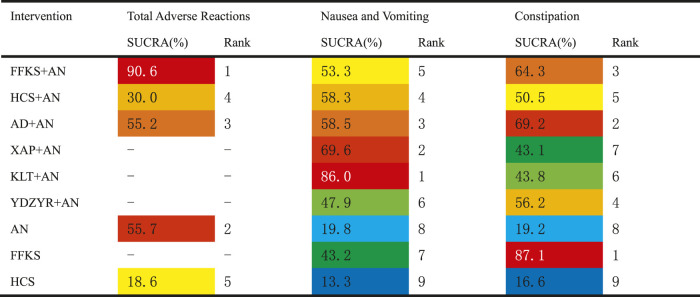

Note: The warmer the color, the greater the SUCRA, and the greater the probability of becoming the best intervention.

FFKS + AN, Fufangkushen injection + analgesic; HCS + AN, Huachansu injection + analgesic; AD + AN, Aidi injection + analgesic; XAP + AN, Xiaoaiping injection + analgesic; KLT + AN, Kanglaite injection + analgesic; YDZYR + AN, Yadanziyouru injection + analgesic; AN, analgesic; FFKS, fufangkushen injection; HCS, huachansu injection.

##### Nausea and Vomiting

Forty-nine studies clearly reported the number of patients with nausea and vomiting, involving nine interventions (FFKS + AN, HCS + AN, AD + AN, XAP + AN, KLT + AN, YDZYR + AN, AN, FFKS, HCS), and eight pairs direct comparisons were generated. The network relationships among the interventions were shown in Figure 3.

There were 36 pairs comparisons in terms of the incidence of nausea and vomiting, and four indicated statistically significant differences. Compared with HCS (OR = 1.93, 95% CI [1.02–3.65]) (OR = 3.26, 95% CI [1.46–7.26]) and AN (OR = 1.65, 95% CI [1.34–2.03]) (OR = 3.80, 95% CI [1.39–10.37]), FFKS + AN and KLT + AN were safer.

Ranking the nine interventions based on the SUCRA values, the results were as follows: KLT + AN (86%), XAP + AN (69.6%), AD + AN (58.5%), HCS + AN (58.3%), FFKS + AN (58.3%), YDZYR + AN (47.9%), FFKS (43.2%), AN (19.8%), and HCS (13.3%), as shown in Figure 4 and Table 5.

##### Constipation

A total of 45 studies reported the number of patients with constipation, which constituted eight pairs of direct comparisons (no closed loop), involving six types of TCMIs and nine interventions (FFKS + AN, HCS + AN, AD + AN, XAP + AN, KLT + AN, YDZYR + AN, AN, FFKS, HCS). The above results were detailed in Figure 3.

There were 36 pairs comparisons in the NMA in terms of the incidence of constipation, and two indicated statistically significant differences. Compared with AN (OR = 1.91, 95% CI [1.57–2.32]) and HCS (OR = 2.06 [1.11–3.80]), FFKS + AN was safer.

Ranking of nine interventions based on SUCRA values, the results were as follows: FFKS (87.1%), AD + AN (69.2%), FFKS + AN (64.3%), YDZYR + AN (56.2%), HCS + AN (50.5%), KLT + AN (43.8%), XAP + AN (43.1%), AN (19.2%), and HCS (16.6%). Specific values were shown in Table 5.

### 3.5 Radar Presentation and Bubble Diagram

**FIGURE 5:**
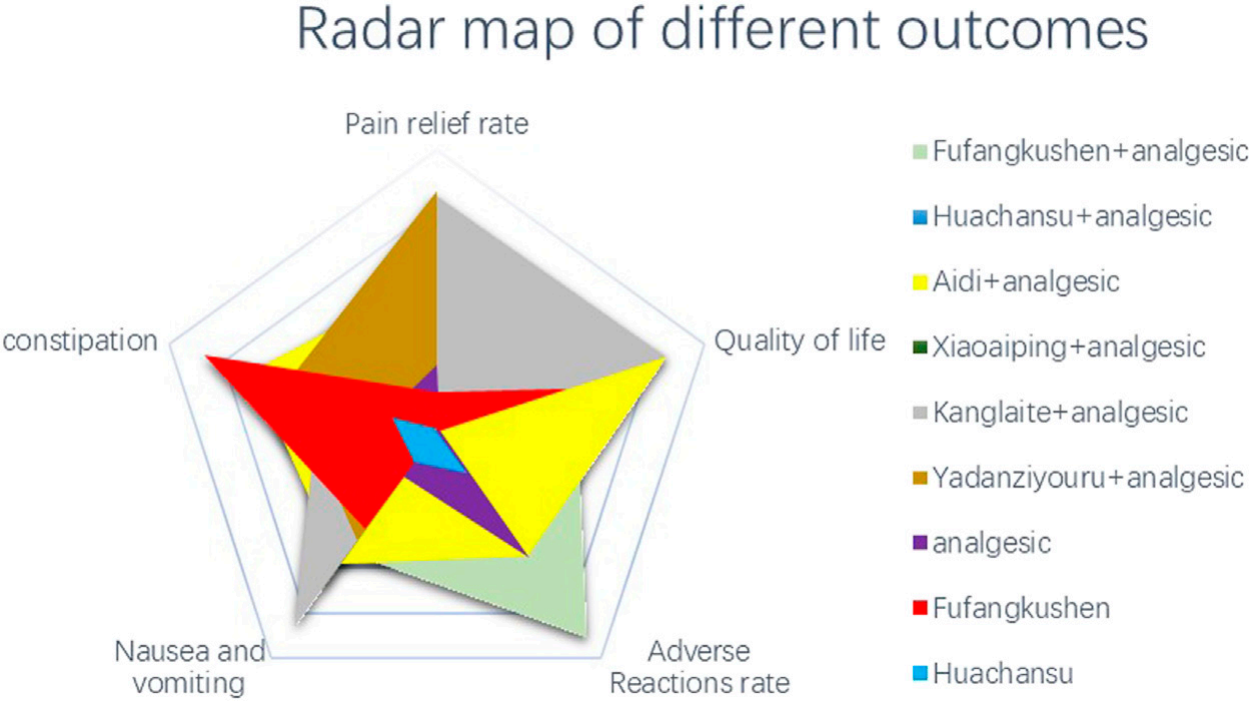
Radar map of different outcomes. Note: the outstanding interventions appear on the outermost side of the corresponding line in the radar map.

We performed a pictorial presentation for the five outcomes *via* a radar map based on the SUCRA results. We found YDZYR + AN (85.3%) was the most effective regimen taking account of pain relief rate, followed by KLT + AN (83.6%). In terms of improving quality of life, AD + AN (86.2%) was the best treatment, followed by KLT + AN (85.7%). The total incidence of adverse reactions of FFKS + AN (90.6%) was the lowest, and the constipation rate of FFKS (87.1%) was the lowest. The incidence of nausea and vomiting of KLT + AN (86.0%) was the lowest. (Figure 5).

**FIGURE 6:**
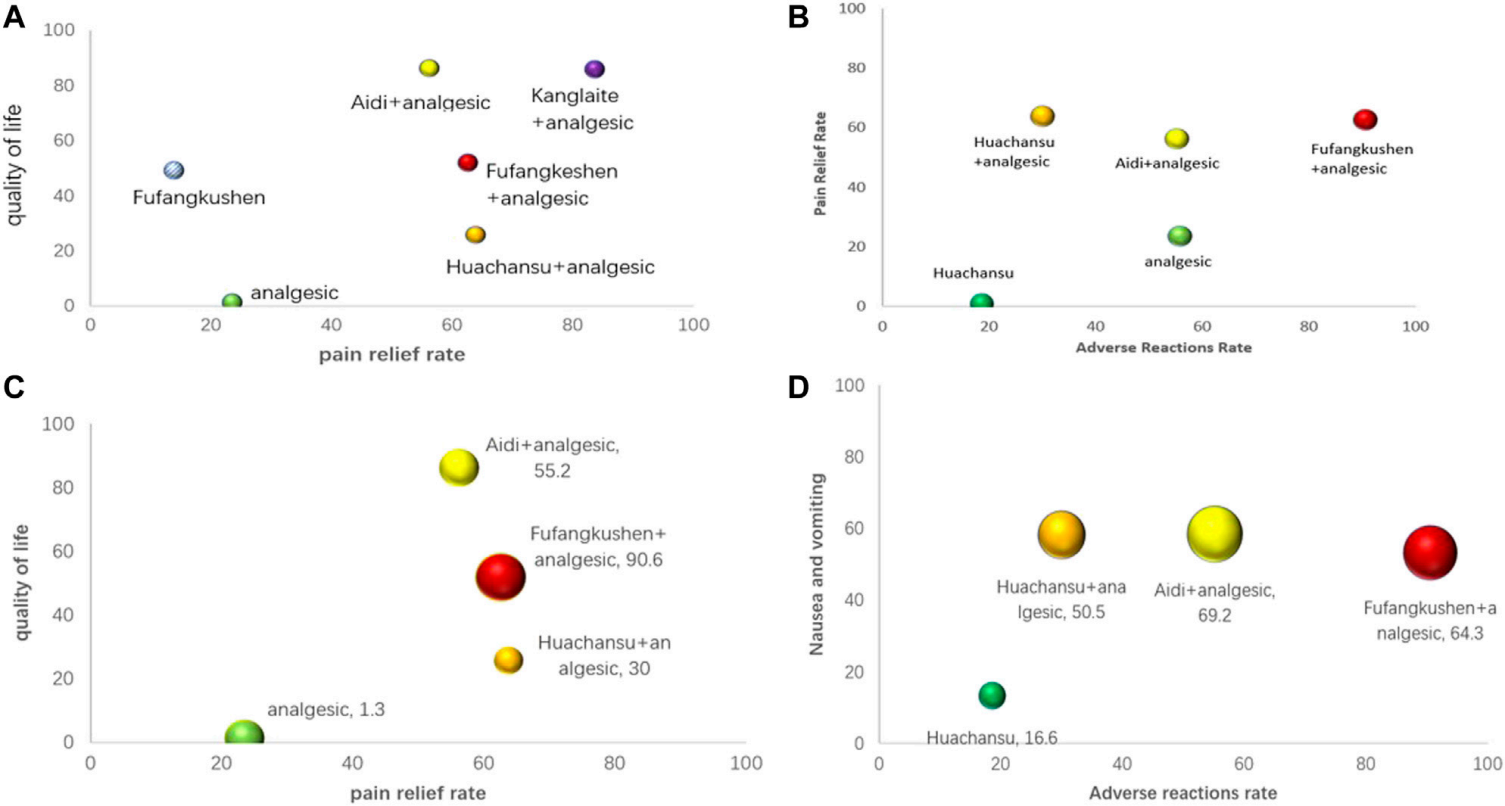
Synthetic sorting bubble diagram plot for outcomes. **(A)** bubble diagram plot for pain relief rate and quality of life;**(B)** bubble diagram plot for pain relief rate and total adverse reactions rate; **(C)** bubble diagram plot for pain relief rate, quality of life and total adverse reactions rate; **(D)** bubble diagram plot for the incidence of total adverse reactions, nausea and vomiting and constipation. Note: Interventions with the same color belonged to the same regimen, and interventions located in the upper right corner indicate optimal therapy for two different outcomes. The bubble area sizes in C and D represent the third dimension’s outcome.

Also, we used synthetic sorting bubble diagrams to comprehensively present the relative better intervention for CRP in this NMA. Bubble plots indicated that taking account of pain relief rate and total incidence of adverse reactions, FFKS + AN was the preferred treatment. Simultaneously, it was the regimen with lowest incidence of constipation, nausea and vomiting, and total adverse reactions. KLT + AN was the best in alleviating pain and improving quality of life (Figure 6).

### 3.6 Publication Bias and Sensitivity Analysis

**FIGURE 7:**
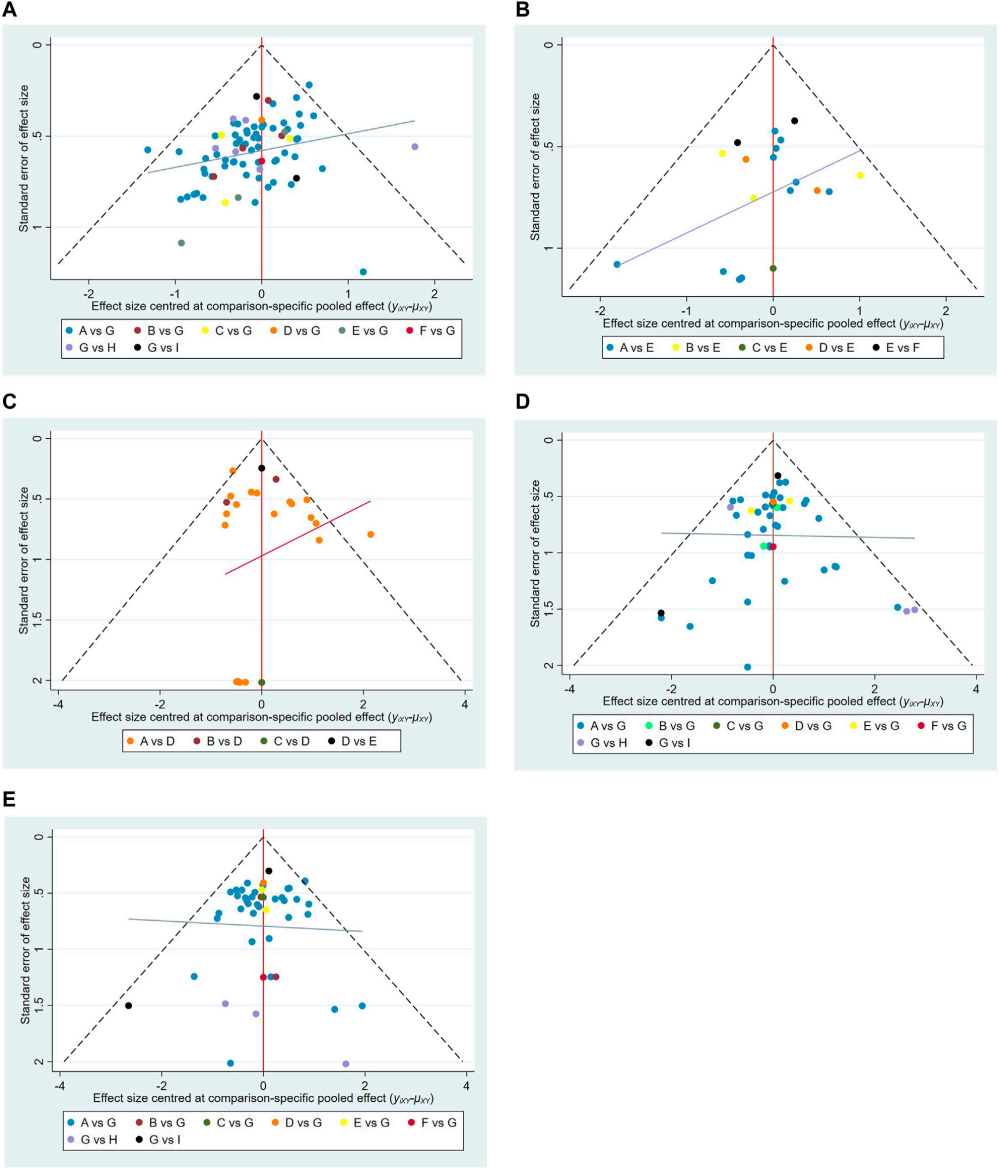
Funnel plots. **(A)** Pain relief rate; **(B)** KPS; **(C)** Total Adverse Reactions; **(D)** Nausea and vomiting; **(E)** constipation. Note: A,Fufangkushen injection+analgesic; B,Huachansu injection+analgesic; C,Aidi injection + analgesic; D,Xiaoaiping injection+analgesic; E,Kanglaite injection+analgesic; F,Yadanziyouru injection+analgesic; G,analgesic; H, Fufangkushen injection; I, Huachansu injection.

Publication bias was detected *via* comparison-adjusted funnel plots for five outcomes respectively. It had poor symmetry in pain relief rate (Egger test: t = −4.15, *p* = 0.001 < 0.05; Begg test: Z = 3.84, *p* = 0.001 < 0.05) and quality of life (Egger test: t = −2.56, *p* = 0.019 < 0.05; Begg test: Z = 0.68, *p* = 0.496 > 0.05), indicating that there was some potential publication bias in the included studies, which may be caused by small sample effects. The results showed there were unobvious publication bias in the total incidence of adverse reactions (Egger test: t = 0.66, *p* = 0.516 > 0.05; Begg test: Z = 0.91, *p* = 0.362 > 0.05), nausea and vomiting (Egger test: t = −0.02, *p* = 0.988 > 0.05; Begg test: Z = 0.71, *p* = 0.475 > 0.05), and constipation (Egger test: t = −1.02, *p* = 0.312 > 0.05); Begg test: Z = 0.01, *p* = 0.993 > 0.05), as shown in Figure 7.

Also, sensitivity analysis was performed by excluding each RCT individually from the present study and the results were relatively robust (Supplementary Material S6).

## 4 Discussion

This NMA incorporated 84 RCTs, comparing the efficacy and safety of nine interventions for CRP. To our knowledge, this is the first study that compare the efficacy and safety of TCMIs regimens, which considers all TCMIs approved for marketing by NMPA. We had two main findings. Firstly, FFKS + AN was the regimen with lowest adverse reaction rate and highest pain relief rate. Moreover, it was the safest intervention. KLT + AN was the most appropriate regimen in relieving pain and improving quality of life. Secondly, compared with analgesic alone, TCMIs + AN regimens were considered as a favorable choice in improving pain relief rate and quality of life. Simultaneously, the incidence of nausea and vomiting as well as constipation was the lowest.

When it comes to efficacy, we found that YDZYR + AN ranked first when only taking account of pain relief rate, KLT + AN following closely. In terms of quality of life, AD + AN was the best treatment, followed by KLT + AN closely. Comprehensively, KLT + AN was the best in alleviating pain and improving quality of life simultaneously. The analgesic effects of KLT may be related to the reduction of pro-inflammatory cytokines TNF-α and IL-Iβ and increase of pain threshold (Tan et al., 2007). Previous studies have proved that KLT plus chemotherapy could relieve pain and improve quality of life (Ding et al., 2020; Liao et al., 2017; Liu et al., 2016), but KLT + AN still lacks high-quality RCT evidence. Moreover, our study showed that TCMIs + AN was more effective than AN, which is consistent with previous researches (Lv et al., 2020). Interestingly, we found that FFKS is better than HCS + AN in improving quality of life. This conclusion still needs further researches to confirm considering the quality, number, and baseline characteristics of RCTs included. For example, the different proportion of patients with mild, medium, and severe pain in each RCT and the inclusion of some small sample studies may underestimate or overestimate the effect of HCS + AN.

As for safety, bubble diagrams showed that FFKS + AN is the safest regimen, which is similar to the previous studies (Ma et al., 2018). However, HCS ranked last in terms of all safety indicators and HCS + AN ranked middle interestingly. Considering the quality of RCTs included, it still needs more high-quality RCTs to prove their safety. Meanwhile, previous studies mainly showed that TCMIs + AN could achieve lower adverse reactions, which is partly consistent with our research (Lv et al., 2020). For instance, we found the incidence of constipation of FFKS was lower than TCMIs + AN (FFKS + AN, HCS + AN, AD + AN, XAP + AN, KLT + AN, YDZYR + AN) and the incidence of total adverse reactions of AN was lower than HCS + AN or AD + AN. The adverse reactions of TCMIs + AN is higher than TCMIs. It can be speculated the adverse reactions of AN itself (Manuel et al., 2021).

Integrating the total adverse reaction rate and pain relief rate, FFKS + AN was the best regimen, which is similar with the previous studies. A meta-analysis involving 15 trials showed that compared with opioid, FFKS plus opioid could improve the pain relief rate and the adverse reaction rate was lower, such as nausea, drowsiness, and constipation (Ma et al., 2018). Another study indicated that KKFS plus zoledronic acid had higher clinical efficacy in relieving bone cancer pain and the adverse reaction rate was not increased significantly (Chen et al., 2019). The analgesic effects of FFKS may be associated with the transmembrane influx of Ca^2+^ and the output of NO (Luo et al., 2001; Gao et al., 2018).

Several limitations are worth mentioning. Firstly, all of the included RCTs were conducted in China, which leads to a regional publication bias to a certain extent. This would limit the extrapolation of our conclusions. Secondly, according to the result of risk of bias evaluation, we found that the quality of included RCTs was generally not very good, especially the implementation of the blind method and allocation concealment. These deficiencies in clinical trial design will directly affect the quality of original RCTs, thus posing a challenge to the quality of our secondary research. Thirdly, the imbalance of study characteristics between nine interventions may affect our conclusions. For instance, the imbalanced number of RCT regarding each intervention, small sample studies, different primary tumor types, disease stages, and different analgesics may cause confounding bias to our research. Particularly, different scales (NRS, VAS, and VRS) were used to measure pain intensity, which lack internationally recognized way to integrate these results. Lastly, the lack of detailed reports of adverse reactions may affect the reliability of safety results. It is worth mentioning that only four RCTs (He et al., 2014; Xu et al., 2011; Fu et al., 2012; Cai et al., 2013) reported the reasons for withdrawal and loss of follow-up in detail (due to adverse reactions of OxyContin). Given the above limitations, it is recommended that more high-quality researches should be performed as perfectly as possible to ensure the reliability of our conclusions.

## 5 Conclusion

In conclusion, FFKS + AN was the best regimen considering the integrated outcomes of the total adverse reaction rate and pain relief rate. Also, it was the safest regimen. KLT + AN may be the best choice for relieving pain and improving the quality of life. TCMIs + AN regimens are superior to AN regimen in improving efficacy. More large sample sizes and high-quality RCTs are wanted to confirm and support this NMA (Qu, 2017; Science Popularization Department of Chinese Anti-Cancer Association, 2020).

## Data Availability

The original contributions presented in the study are included in the article/Supplementary Material. Further inquiries can be directed to the corresponding author.

## Abbreviations

AD + AN, Aidi injection plus analgesic; AN, Analgesic; 95% CIs, 95% confidence intervals; CRP, Cancer-related pain; FFKS, Fufangkushen injection; FFKS + AN, Fufangkushen injection plus analgesic; HCS, Huachansu injection; HCS + AN, Huachansu injection plus analgesic; KLT + AN, Kanglaite injection plus analgesic; KPS, Karnofsky Performance Score; MD, Mean Differences; NMA, network meta-analysis; NRS, Numerical Rating Scale; OR, Odds Ratio; RCTs, Randomized controlled trials; SUCRA, The surface under the cumulative ranking curve; TCMIs, Traditional Chinese Medicine Injections; VAS, Visual Analogue Scale; VRS, Verbal Rating Scale; XAP + AN, Xiaoaiping injection plus analgesic; YDZYR + AN, Yadanziyouru injection plus analgesic.
