# Supplementary material for: Comparative Analysis of the Efficacy and Safety of Different Traditional Chinese Medicine Injections in the Treatment of Cancer-Related Pain: A Bayesian Network Meta-Analysis
Source: Front Pharmacol. 2022 Feb 7;12:803676. doi: 10.3389/fphar.2021.803676 (PMC8858830; doi:10.3389/fphar.2021.803676)
Supplement: Supplementary file 2 [file DataSheet1.docx]

File 1

TCMIs met inclusion and exclusion criteria (n=6)

Xiaoaiping injection; Hanchansu injection; Aidi injection; Kanglaite injection; Fufangkeshen injection; Yadanziyouru injection

TCMIs mentioned the indications of cancer or cancer pain in their drug instructions (n=22)

Chansu injection; Fufangkeshen injection; Hanchansu injection; Kanglaite injection; Aidi injection; Xiaoaiping injection; Yadanziyouru injection; Kangai injection; Coicisoil injection; Yuanqinzhitong injection; Yitongshu injection; Xiangguduotang injection; Tongan injection; Ginseng polysaccharide injection; Jishiteng injection; Fufangbanbianlian injection; Shenqifuzheng injection; Baihuasheshecao injection; Zhulingduotang injection; Huangqiduotang injection; Zhongjiefeng injection; Wutou injection

Traditional Chinese Medicine Injections (TCMIs) approved by NMPA (n=135)

File 2

PRISMA checklist (Separate document)

File 3

Search strategies

CNKI

**#1**: (题名=复方苦参注射液 OR 题名=岩舒注射液 OR 题名=复方苦参注射剂) OR (主题=复方苦参注射液 OR 主题=岩舒注射液 OR 主题=复方苦参注射剂)

#2: (题名=蟾酥注射液 或者 题名=蟾酥注射剂 OR 题名=佳素 OR 题名=史君轻) OR (主题=蟾酥注射液 OR 主题=蟾酥注射剂 OR 主题=佳素 OR 主题=史君轻)

#3: (题名 =注射用薏苡仁油) OR (主题=注射用薏苡仁油)

#4: (题名=元秦止痛注射液 OR 题名=元秦止痛注射剂) OR (主题=元秦止痛注射液 OR 主题=元秦止痛注射剂)

#5: (题名=鸦胆子油乳注射液 OR 题名=鸦胆子油乳注射剂) OR (主题=鸦胆子油乳注射液 OR 主题=鸦胆子油乳注射剂)

#6: (题名=消癌平注射液 OR 题名=消癌平注射剂 OR 题名=通关藤注射液) OR (主题=消癌平注射液 OR 主题=消癌平注射剂 OR 主题=通关藤注射液)

#7: (题名=人参多糖注射液 或者 题名=人参多糖注射剂 或者 题名=安尔欣 或者 题名=奥康莱 或者 题名=癌得安 或者 题名=百扶欣) OR (主题=人参多糖注射液 或者 主题=人参多糖注射剂 或者 主题=安尔欣 或者 主题=奥康莱 或者 主题=癌得安 或者 主题=百扶欣)

#8: (题名=华蟾素注射液 或者 题名=华蟾素注射剂 ) OR (主题=华蟾素注射液 或者 主题=华蟾素注射剂 )

#9: (题名=注射用艾迪 或者 题名=艾迪液 或者 题名=艾迪注射液 或者 题名=艾迪注射剂 或者 题名=爱迪注射液 或者 题名=爱迪注射剂 ) OR (主题=注射用艾迪 或者 主题 =艾迪液 或者 主题=艾迪注射液 或者 主题 =艾迪注射剂 或者 主题=爱迪注射液 或者 主题=爱迪注射剂)

#10: (题名 =康莱特注射液 或者 题名 =康莱特注射剂 或者 题名 =ZCE-3静脉乳 ) OR (主题=康莱特注射液 或者 主题=康莱特注射剂 或者 主题=ZCE-3静脉乳)

#11: (题名=康艾液 或者 题名=康艾注射液 或者 题名=康艾注射剂 ) OR ( 主题=康艾液 或者 主题=康艾注射液 或者 主题=康艾注射剂)

#12: (题名=参芪扶正注射液 或者 题名=参芪扶正注射剂) OR ( 主题=参芪扶正注射液 或者 主题=参芪扶正注射剂)

#13: (题名=猪苓多糖注射液 或者 题名=猪苓多糖注射剂) OR ( 主题=猪苓多糖注射液 或者 主题=猪苓多糖注射剂)

#14: (题名=注射用黄芪多糖 或者 题名=黄芪多糖注射液 或者 题名=黄芪多糖注射剂 ) OR (主题=注射用黄芪多糖 或者 主题=黄芪多糖注射液 或者 主题=黄芪多糖注射剂)

#15: (题名=乌头注射液 或者 题名=泰癌注射液 或者 题名=乌头注射剂 ) OR (主题=乌头注射液 或者 主题=泰癌注射液 或者 主题=乌头注射剂 ) ) )

#16: (题名=伊痛舒注射液 或者 题名=伊痛舒注射剂 ) OR (主题 =伊痛舒注射液 或者 主题 =伊痛舒注射剂)

#17: (题名=香菇多糖注射液 或者 题名=香菇多糖注射剂 ) OR (主题=香菇多糖注射液 或者 主题=香菇多糖注射剂)

#18: (题名=痛安注射液 或者 题名=痛安注射剂 ) OR ( 主题=痛安注射液 或者 主题=痛安注射液剂)

#19: (题名=鸡矢藤注射液 或者 题名=鸡矢藤注射剂 ) 或者 (主题=鸡矢藤注射液 或者 主题=鸡矢藤注射剂)

#20: (题名=复方半边莲注射液 或者 题名=复方半边莲注射剂 ) 或者 (主题=复方半边莲注射液 或者 主题=复方半边莲注射液剂)

#21: (题名=白花蛇舌草注射液 或者 题名=白花蛇舌草注射剂 ) 或者 ( 主题=白花蛇舌草注射液 或者 主题=白花蛇舌草注射剂)

#22: ( 题名=肿节风注射液 或者 题名=肿节风注射剂 ) 或者 ( 主题=肿节风注射液 或者 主题 =肿节风注射剂) (精确匹配)

#23: #1 OR #2 OR #3 OR#4 OR #5 OR #6 OR #7 OR #8 OR #9 OR #10 OR #11 OR #12 OR #13 OR #14 OR #15 OR #16 OR #17 OR #18 OR #19 OR #20 OR #21 OR #22

#24: (题名=癌痛 OR 题名=癌性疼痛 OR 题名=癌症疼痛) OR (主题=癌痛 OR 主题=癌性疼痛 OR 主题=癌症疼痛)

#25: (AB=随机 OR AB=RCT OR AB=随机对照 OR AB=随机分组

#26: #23 AND #24 AND #25

PUBMED:

#1

Search: (venenum bufonis liposome injection[Title/Abstract]) or (ChanSu injection[Title/Abstract]) or (secretio bufonis injection[Title/Abstract]) or (coix seed oil injection[Title/Abstract]) or (coicis oil injection[Title/Abstract]) or (Semen Coicis Yokuinin injection[Title/Abstract]) or (yuanqinzhitong injection[Title/Abstract]) or (yitongshu injection[Title/Abstract]) or (yadanziyou injection [Title/Abstract]) or (yadanzi oil injection[Title/Abstract]) or (fructus bruccae emulsion [Title/Abstract]) or (brucei javanica oil emulsion injection[Title/Abstract]) or (fructus bruceae emulsion injection[Title/Abstract]) or (xiaoaiping injection[Title/Abstract]) or (xiangguduotang injection[Title/Abstract]) or (lentinas[Title/Abstract]) or (tongan injection[Title/Abstract]) or (ginseng polysaccharide injection[Title/Abstract]) or (renshen duotang injection[Title/Abstract]) or (Huachansu injection[Title/Abstract]) or (Cinobufacini[Title/Abstract]) or (aidi injection[Title/Abstract]) or (Kanglaite injection[Title/Abstract]) or (kangai injection[Title/Abstract]) or (paederia scandens injection[Title/Abstract]) or (jishiteng injection[Title/Abstract]) or (fufangkushen injection[Title/Abstract]) or (compound kushen injection[Title/Abstract]) or (compound radix sophorae flavescentis injection[Title/Abstract]) or (yanshu injection[Title/Abstract]) or (fufang banbianlian injection[Title/Abstract]) or (shenqi fuzheng injection[Title/Abstract]) or (baihuasheshecao injection[Title/Abstract]) or (hedyotis dffusa willd injection[Title/Abstract]) or (zhuling polysaccharide injection[Title/Abstract]) or (injection with PPS[Title/Abstract]) or (polyporus polysaccharide injection [Title/Abstract]) or (huangqi fuzheng injection[Title/Abstract]) or (zhongjiefeng injection [Title/Abstract]) or (sarcandrae injection[Title/Abstract]) or (wutou injection[Title/Abstract]) or (aconite injection[Title/Abstract]) Sort by: Publication Date

#2

Search: (cancer pain[Title/Abstract]) OR (Cancer Pains[Title/Abstract]) OR(Pain, Cancer[Title/Abstract]) OR(Pains, Cancer[Title/Abstract]) OR(Cancer-Associated Pain[Title/Abstract]) OR(Cancer Associated Pain[Title/Abstract]) OR(Cancer-Associated Pains[Title/Abstract]) OR(Pain, Cancer-Associated[Title/Abstract]) OR(Pains, Cancer-Associated[Title/Abstract]) OR(Neoplasm-Related Pain[Title/Abstract]) OR(Neoplasm Related Pain[Title/Abstract]) OR(Neoplasm-Related Pains[Title/Abstract]) OR(Pain, Neoplasm-Related[Title/Abstract]) OR(Pains, Neoplasm-Related[Title/Abstract]) OR(Oncological Pain[Title/Abstract]) OR(Oncological Pains[Title/Abstract]) OR(Pain, Oncological[Title/Abstract]) OR(Pains, Oncological[Title/Abstract]) OR(Tumor-Related Pain[Title/Abstract]) OR(Pain, Tumor-Related[Title/Abstract]) OR(Pains, Tumor-Related[Title/Abstract]) OR(Tumor Related Pain[Title/Abstract]) OR(Tumor-Related Pains[Title/Abstract]) OR(Tumor-Associated Pain[Title/Abstract]) OR(Pain, Tumor-Associated[Title/Abstract]) OR(Pains, Tumor-Associated[Title/Abstract]) OR(Tumor Associated Pain[Title/Abstract]) OR(Tumor-Associated Pains[Title/Abstract]) OR(Oncology Pain[Title/Abstract]) OR(Oncology Pains[Title/Abstract]) OR(Pain, Oncology[Title/Abstract]) OR(Pains, Oncology[Title/Abstract]) OR(Cancer-Related Pain[Title/Abstract]) OR(Cancer Related Pain[Title/Abstract]) OR(Cancer-Related Pains[Title/Abstract]) OR(Pain, Cancer-Related[Title/Abstract]) OR(Pains, Cancer-Related[Title/Abstract]) OR(Neoplasm-Associated Pain[Title/Abstract]) OR(Neoplasm Associated Pain[Title/Abstract]) OR(Neoplasm-Associated Pains[Title/Abstract]) OR(Pain, Neoplasm-Associated[Title/Abstract]) OR(Pains, Neoplasm-Associated[Title/Abstract]) Sort by: Publication Date

#3

Search: (randomized controlled trial [Publication Type] OR randomized [Title/Abstract] OR placebo [Title/Abstract]) Sort by: Publication Date

#4: #1 and #2 and #3 （Filters: Clinical Trial or RCT Sort by: Publication Date）

File 4

More details about the product information of 6 TCMIs

| TCMIs name | Raw materials | Labeled efficacy | Labeled adverse reactions | Indications |
| --- | --- | --- | --- | --- |
| Fufangkushen injection | Sophorae Flavescentis  Radix, Smilacis Glabrae  Rhizoma | Clearing away hot, removing dampness, cooling blood, removing toxin, resolving hard mass, and relieving pain | 1.Occasionally nausea, vomiting, fever, chills, abdominal distension and stomach discomfort and other symptoms.  2.Occasional allergic reactions, manifested as head and neck skin flushing, sweating, rash, pruritus, etc., may be related to the patient's specific constitution. Topical use is mildly irritating but well absorbed. | Cancer pain, bleeding |
| Huachansu injection | Bufonis Corium | Removing toxin, detumescence, and relieving pain | 1.For individual patients, if the dosage is too large or the interval between two drugs is less than 6 ~ 8 hours, about 30 minutes after the drug, the phenomenon of chills and fever may appear;  2.A small number of patients after long-term intravenous drip local irritation or phlebitis, resulting in slow drip rate, very individual patients may also appear urticaria, dermatitis, etc. | Middle and advanced cancer, chronic hepatitis B etc.. |
| Aidi injection | Mylabris, Ginseng Radix Et Rhizoma, Astragali Radix, Acanthopanacis Senticosi Radix Et Rhizoma Seu Caulis | Clearing away hot, removing toxin, resolving stagnation and dispersing masses | 1.After the first application of this product, some patients occasionally experienced red face, urticaria, fever and other reactions, and very few patients had palpitations, chest tightness, nausea and other reactions. | Primary liver cancer, lung cancer, rectal cancer, malignant lymphoma, gynecological malignant tumors, etc. |
| Xiaoaiping injection | Marsdenia tenacissima Caul | Clearing away heat and toxic material, resolving phlegmand softening hard masses | 1.Allergic reactions: systemic skin flushing, rash, pruritus, dyspnea, palpitation, cyanosis, decreased blood pressure, laryngeal edema, anaphylactic shock, etc.  2.musculoskeletal: migratory myalgia, joint pain, etc.  3.systemic reaction: fever, chills, pain, fatigue and so on.  4.skin and accessories: rash, itching, sweating, etc.  5.Digestive system: nausea, vomiting, abdominal pain, diarrhea, etc.  6.Respiratory system: dyspnea, cough, etc.  7.Cardiovascular system: chest tightness, palpitation, elevated or decreased blood pressure, etc.  8.nervous system: dizziness, headache and so on.  9.Others: pain at the injection site, phlebitis, etc. | Esophageal cancer, gastric cancer, lung cancer, liver cancer; combination with radiotherapy and chemotherapy as adjuvant therapy |
| Kanglaite injection | Jobstears Seed Oil | Tonifying Qi, nourishing Yin, and resolving mass | 1.Lipid allergy is occasionally seen in clinic, such as chills, fever, mild nausea and liver transaminase reversible increase. Most of these symptoms can disappear naturally and adapt after 3-5 days of use.  2.Slight phlebitis is occasionally seen. | Unfavorable operation of Qi and yin deficiency, spleen deficiency and dampness retention with primary non-small cell lung cancer and primary liver cancer; has anti cachexia and analgesic effects in patients with advanced cancer |
| Yadanziyouru injection | Bruceae Fructus | Anticarcinogen | 1.Allergic reactions: flushing, rash, pruritus, dyspnea, palpitations, cyanosis, decreased blood pressure, anaphylactic shock, etc.  2.Systemic reaction: chills, chills, fever, sweating, etc.  3.Digestive system: nausea, vomiting, abdominal pain, greasy feeling, anorexia, abnormal liver biochemical indicators.  4.Respiratory system: chest tightness, suffocation, dyspnea, etc.  5.Skin and accessories: rash, pruritus, etc.  6.Cardiovascular system: palpitation, flushing, and occasional death from arrhythmia.  7.Nervous system: dizziness, headache, convulsions, etc.  Others: Phlebitis. | Lung cancer, brain metastasis of lung cancer and digestive system neoplasms |

File 5

| **Study ID** | **Types of cancer** |
| --- | --- |
| LIU A 2005 | Lung cancer, breast cancer, digestive tract cancer, bladder cancer |
| LIU YX 2005 | Lung cancer, breast cancer, stomach cancer, primary liver cancer, pancreatic cancer, colon cancer, ovarian cancer, bladder cancer |
| ZOU JF 2006 | Lung cancer, liver cancer, stomach cancer, bowel cancer, other visceral metastasis cancer and soft tissue metastasis cancer |
| CHEN SQ 2007 | Lung cancer, stomach cancer, breast cancer, liver cancer, rectum cancer, cervix cancer, prostate cancer, esophageal cancer, ovarian cancer, malignant lymphoma cancer, brain tumor, or distant metastases cancer |
| MA YY 2008 | Lung cancer, stomach cancer, breast cancer, liver cancer, rectal cancer, cervical cancer, prostate cancer, esophageal cancer, ovarian cancer, malignant lymphoma, brain tumor, complicated visceral metastasis and soft tissue metastasis |
| SI C 2008 | Gastric cancer, liver cancer, esophageal cancer, colorectal cancer, peritoneal metastatic cancer, peritoneal primary cancer, pancreatic cancer, primary gastric malignant lymphoma |
| YANG YX 2009 | Lung cancer, esophageal cancer, nasopharyngeal carcinoma, gastric cancer, breast cancer, liver cancer, colorectal cancer, soft tissue tumor, malignant lymphoma, pancreatic cancer, ovarian cancer, cervical cancer, laryngeal cancer, kidney cancer and multiple myeloma, prostate cancer, oral cancer, bladder cancer, testicular cancer, tongue cancer, skin cancer, kidney cancer, endometrial cancer, malignant pleural mesothelioma, metastatic carcinoma other unknown reasons |
| CHENG JD 2009 | Lung cancer, colon cancer, breast cancer, liver cancer, stomach cancer |
| SU QS 2009 | Gastric cancer, bowel cancer, liver cancer |
| LIU JL 2010 | Liver cancer, stomach cancer, colon cancer, and metastatic cancer |
| PAN CS 2010 | Liver cancer, stomach cancer, colon cancer, and metastatic cancer |
| CHEN Y 2011 | Gastric cancer, intestinal cancer, liver cancer |
| LANG J 2011 | Gastric cancer, intestinal cancer, liver cancer |
| XU CA 2011 | Lung cancer |
| LIN MX 2011 | Lung cancer, liver cancer, colorectal cancer, breast cancer, pancreatic cancer, nasopharyngeal cancer, esophageal cancer and their metastatic cancer |
| GUAN NB 2011 | Lung cancer, stomach cancer, breast cancer, rectal cancer, liver cancer, cervical cancer, prostate cancer, esophageal cancer, ovarian cancer, malignant lymphoma, brain tumor and their metastatic cancer |
| FU Y 2012 | Lung cancer, liver cancer, breast cancer, stomach cancer, rectum cancer, nasopharyngeal cancer, cervical cancer, prostate cancer, brain tumor, esophageal cancer, ovarian cancer, malignant lymphoma, gallbladder cancer, pancreatic cancer and their metastatic cancer |
| DOU LH 2012 | Stomach cancer, esophageal cancer, breast cancer |
| CHEN YZ 2012 | - |
| YANG JL 2012 | - |
| ZENG L 2012 | Lung adenocarcinoma, lung squamous cell carcinoma, lung small cell carcinoma, adenosquamous carcinoma |
| MING XH 2013 | Liver cancer, stomach cancer, colon cancer and metastatic cancer |
| HUANG KQ 2013 | Stomach cancer |
| ZHAO YH 2013 | - |
| ZHAO CH 2013 | Lung cancer, liver cancer, breast cancer, stomach cancer, rectum cancer, esophagus cancer, ovarian cancer, pancreatic cancer, gallbladder cancer, cervical cancer, bladder cancer |
| YAO B 2013 | Lung cancer, stomach cancer, bowel cancer, liver cancer, breast cancer, pancreatic cancer |
| DAI GH 2013 | Lung cancer, colorectal cancer, breast cancer, stomach cancer, liver cancer, nasopharyngeal cancer, cervical cancer, esophageal cancer, ovarian cancer and their metastatic cancer |
| WANG L 2013 | Stomach cancer, esophageal cancer, breast cancer |
| QI HX 2013 | Gastric cancer, bowel cancer, liver cancer |
| CAI ZH 2013 | Lung cancer, stomach cancer, liver cancer, esophageal cancer, pancreatic cancer, osteosarcoma, colorectal cancer, breast cancer |
| ZHAO J 2013 | Lung cancer, breast cancer, stomach cancer, colorectal cancer, esophageal cancer, pancreatic cancer, prostate cancer, liver cancer, pleural mesothelioma |
| XIE DF 2013 | Lung cancer, bowel cancer, breast cancer, liver cancer, stomach cancer |
| LIU M 2014 | Gastric cancer, colorectal cancer, breast cancer, lung cancer, liver cancer |
| WANG DR 2014 | Esophagus cancer, lung cancer, liver cancer, stomach cancer and bowel cancer |
| HUANG KD 2014 | Breast cancer, esophageal cancer, colorectal cancer, liver cancer, lung cancer, ovarian cancer, stomach cancer |
| LI JC 2014 | Lung cancer, stomach cancer, bowel cancer, liver cancer, breast cancer, pancreatic cancer |
| ZHANG HM 2014 | Colorectal cancer, lung cancer, liver cancer, nasopharyngeal cancer, breast cancer, stomach cancer |
| HE DL 2014 | Lung cancer, breast cancer, stomach cancer, liver cancer, esophageal cancer, pancreatic cancer, osteosarcoma, colorectal cancer |
| ZHOU L 2014 | - |
| YANG YX 2014 | Lung cancer, liver cancer, stomach cancer, colon cancer, prostate cancer, kidney cancer, breast cancer, bladder cancer, cervical cancer |
| JIN Z 2014 | All kinds of cancers |
| SUN LW 2014 | Lung cancer, stomach cancer, liver cancer, esophagus, pancreatic cancer |
| LUAN BH 2014 | - |
| ZHANG C 2015 | Lung cancer, liver cancer, stomach cancer, breast cancer, colon cancer |
| ZHAO YH 2015 | Bone metastasis of cancer |
| FENG XM 2015 | Lung cancer, liver cancer, breast cancer, stomach cancer, rectum cancer, esophagus cancer, ovarian cancer, pancreatic cancer, pancreatic cancer, gallbladder cancer, cervical cancer, bladder cancer |
| QU JR 2015 | Esophageal cancer |
| YUAN XS 2015 | Lung cancer, stomach cancer, bowel cancer, liver cancer, breast cancer, pancreatic cancer |
| ZHANG L 2015 | - |
| ZHANG CL 2016 | Lung cancer, bowel cancer, breast cancer, liver cancer, stomach cancer |
| ZHAO L 2016 | Lung cancer, stomach cancer, esophageal cancer, liver cancer, breast cancer, colorectal cancer, cervical cancer, nasopharyngeal cancer |
| WANG JG 2016 | Lung cancer, stomach cancer, breast cancer, liver cancer, pancreatic cancer |
| MO HY 2016 | Lung cancer, stomach cancer, liver cancer, bowel cancer |
| CHANG PJ 2016 | Lung cancer, stomach cancer, breast cancer, liver cancer, bowel cancer, esophageal cancer, pancreatic cancer, cervical cancer |
| ZHAI Z 2017 | Lung cancer, stomach cancer, esophageal cancer, osteosarcoma, colon cancer, esophageal cancer, pancreatic cancer |
| CHEN GQ 2017 | Colorectal cancer, breast cancer, lung cancer, esophageal cancer, liver cancer |
| JIANG N 2017 | - |
| TIAN XH 2017 | Colon cancer, stomach cancer, esophagus cancer, pancreas cancer, lung cancer |
| LEI JL 2017 | Liver cancer, colon cancer, rectum cancer, esophagus cancer, stomach cancer, pancreas cancer |
| LIU YY 2017 | Lung cancer, stomach cancer, esophageal cancer, kidney cancer, breast cancer |
| LIU T 2017 | Lung cancer, stomach cancer, bowel cancer, liver cancer, breast cancer, pancreatic cancer |
| YAN YD 2017 | Stomach cancer |
| YANG Q 2017 | Breast cancer, prostate cancer, thyroid cancer, kidney cancer, stomach cancer, breast cancer, prostate cancer, bladder cancer, lung cancer, malignant melanoma, head and neck cancer, cervical cancer, bowel cancer |
| WANG J 2017 | - |
| LONG L 2017 | Lung cancer, stomach cancer, colorectal cancer, pancreatic cancer, prostate cancer, esophageal cancer, breast cancer, liver cancer, malignant lymphoma, ovarian cancer, multiple myeloma, soft tissue sarcoma, gingival cancer, nasopharyngeal cancer, bile duct cancer, kidney cancer |
| YAN JY 2018 | Stomach cancer |
| WEN Y 2018 | Lung cancer, breast cancer, gastrointestinal malignancy, gynecological malignancy |
| FENG Y 2018 | - |
| HAO JH 2018 | Pancreas cancer, breast cancer, liver cancer, stomach cancer, colon cancer, cervical cancer |
| LI X 2018 | Lung cancer, breast cancer, digestive tract cancer, urinary tract cancer |
| XIA NX 2018 | Lung cancer, stomach cancer, breast cancer, colorectal cancer, esophageal cancer, liver cancer |
| NIE NL 2018 | Lung cancer |
| LUO Y 2018 | Liver cancer |
| REN F 2018 | Bone metastasis of lung cancer |
| ZHANG P 2018 | Lung cancer, liver cancer, stomach cancer, breast cancer, esophageal cancer |
| WANG Y 2018 | Lung cancer, breast cancer, rectal cancer, stomach cancer |
| WEI L 2018 | Liver cancer, stomach cancer, lung cancer, breast cancer |
| LIU K 2019 | Bone metastases in lung, stomach, colorectal, breast, cervical and colorectal cancers |
| DONG Q 2019 | Lung cancer, liver cancer, stomach cancer, breast cancer, nasopharyngeal cancer |
| LONG J 2020 | Stomach cancer |
| JIANG FL 2020 | - |
| LING ZJ 2020 | Stomach cancer |
| FEI XD 2020 | - |
| LI FF 2021 | Gastric cancer metastases to lung, liver, and bone lymph nodes |

File 6 sensitivity analysis results

pain relief rate

Quality of life

Total adverse reaction rate

Nauseing and vomiting

Constipation
